# Formulation, characterization, and efficacy evaluation of testolift: a novel testosterone-enhancing nutraceutical featuring synergistic black ginger methoxyflavones, fenugreek saponins, and zinc methionine *via* InSitu360 technology

**DOI:** 10.1039/d6ra02748b

**Published:** 2026-07-02

**Authors:** Augustine Amalraj, Kaniyath Ramachandran Reshna, Karthik Varma, Ann Mariya Jogy, Preetha Balakrishnan, Sreerag Gopi

**Affiliations:** a NIMP Innovation Hub, Padmalife Nutrition Private Limited Koratty Thrissur - 680 309 Kerala India sreeraggopi@gmail.com; b Innovation Centre, Padmalife Nutrition Private Limited, Mahatma Gandhi University Innovation Foundation Building No. 10/572, Priyadarshini Hills Athirampuzha, Kottayam Kerala 686560 India; c Global Innovation Centre, Molecules Biolabs Private Limited Koratty Thrissur - 680 309 Kerala India; d Atomin Innovations Private Limited Meloor, Thrissur – 680311 Kerala India

## Abstract

Declining testosterone levels are associated with reduced muscle mass, fatigue, and reproductive challenges in men. Although testosterone replacement therapy is available, it is often associated with risks such as cardiovascular complications and endocrine imbalance, highlighting the need for safer alternatives. Nutraceutical approaches offer a promising strategy for supporting endogenous testosterone production. In this study, Testolift, a novel testosterone-supporting nutraceutical formulation, was developed under the Natural Ingredients for Mental and Performance (NIMP) Platform using InSitu360 molecular complexation technology. The formulation integrates fenugreek saponins, black ginger (*Kaempferia parviflora*) polymethoxyflavones, and zinc methionine to achieve synergistic enhancement of testosterone. The formulation was characterized using FT-IR, SEM, TEM, DSC, EDS, and UPLC, confirming the successful integration of bioactives and favorable physicochemical properties. Quantitative analysis verified the presence of 20% saponins, 0.93% polymethoxyflavones, and 1.4% elemental zinc. Morphological studies revealed uniformly dispersed spherical nano- and microspheres with sub-200 nm particles, supporting structural stability and efficient incorporation of bioactives within the matrix. Dynamic light scattering analysis confirmed a mean particle size of 193 nm with a distribution range of 120–260 nm, while the zeta potential of −32.24 mV indicated good colloidal stability. A 180 days stability study demonstrated >95% retention of methoxyflavones, saponins, and zinc across refrigerated (4 ± 2 °C), ambient (25 ± 2 °C), and accelerated (45 ± 2 °C) conditions, confirming excellent compositional and physicochemical stability. In a 42 days *in vivo* experimental study in male rats, Testolift significantly increased serum testosterone by 22.7% compared to 4.1% in controls (*p* < 0.05) without adverse effects. The InSitu360 technology enhanced the physicochemical and compositional stability of the formulation, supporting its robustness and suggesting potential for improved bioavailability, which warrants confirmation in future clinical studies. These results position Testolift as a promising natural testosterone-support formulation with potential applications in male health and muscle endurance.

## Abstract 2

InSitu360 technology was employed to develop Testolift, a testosterone-boosting formulation comprising fenugreek saponins, black ginger methoxyflavones, and zinc methionine, which significantly increased testosterone levels in rats.

## Graphical abstract

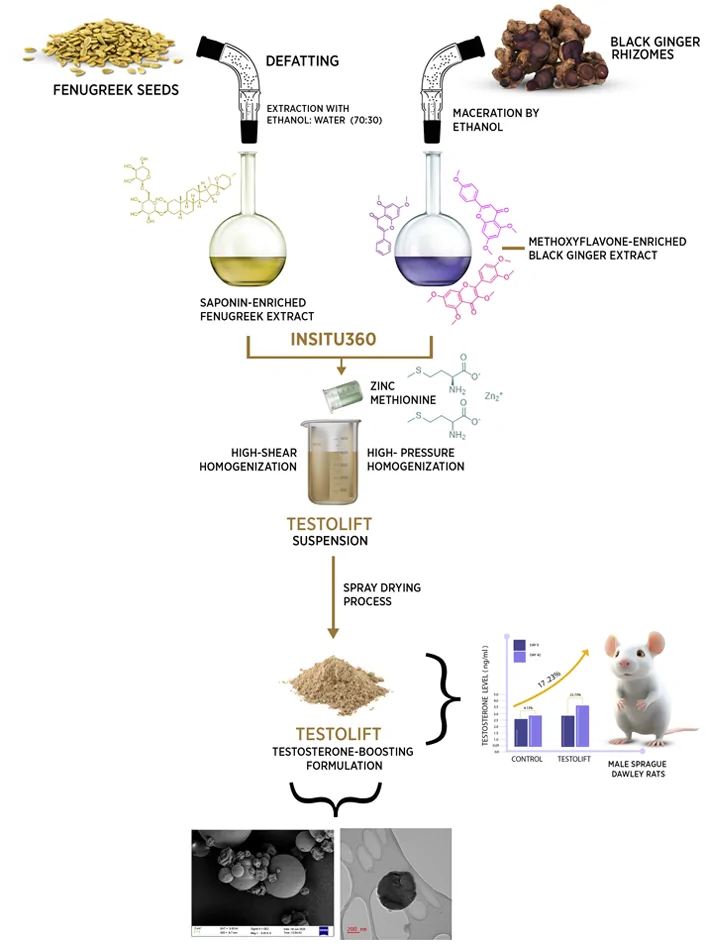

## 1. Introduction

Testosterone is a principal androgen hormone essential for male reproductive function, muscle mass, bone density, skeletal integrity, erythropoiesis, physical stamina, mood regulation, and overall vitality. Declining testosterone levels, whether due to advancing age or lifestyle-related factors such as obesity, chronic disease, and stress, are increasingly recognized as a significant health concern in both older and younger male populations. Symptoms of testosterone deficiency (TD) include diminished libido, fatigue, loss of muscle mass, depression, and cognitive impairment. Global prevalence estimates for TD range from 10% to 40% among adult men, and even mild reductions can adversely affect quality of life and elevate risks for metabolic and cardiovascular disorders. Despite growing awareness, the diagnosis and management of TD remain inconsistent.^1,2^ Although testosterone replacement therapies (TRT) are available, they are associated with drawbacks such as cardiovascular risks, infertility, hepatotoxicity, mood disturbances, and suppression of endogenous testosterone synthesis, potentially leading to dependency and testicular atrophy.^2–4^ These limitations highlight the need for safe and effective alternatives that support natural testosterone production.

Nutraceuticals, defined as products that provide health benefits beyond basic nutrition, have emerged as promising candidates for long-term testosterone support due to their natural origin and favorable safety profile. Unlike synthetic hormones, nutraceutical interventions typically act by supporting the hypothalamic-pituitary-gonadal (HPG) axis, enhancing luteinizing hormone (LH) secretion, reducing oxidative stress, and upregulating steroidogenic enzymes.^2–4^ Current testosterone-supporting nutraceuticals include herbal extracts such as ashwagandha, tongkat ali, tribulus terrestris, and fenugreek, as well as trace minerals like zinc. However, many single-ingredient products suffer from limited clinical validation, poor bioavailability, or insufficient synergy to achieve meaningful improvements in testosterone status and muscle performance.^5–13^ Advanced formulation technologies that combine multiple bioactives and enhance absorption may therefore offer significant advantages.

Testolift is a novel testosterone-boosting nutraceutical formulation that integrates saponin-rich fenugreek (*Trigonella foenum-graecum*) extract, polymethoxyflavone-rich black ginger (*Kaempferia parviflora*) extract, and zinc methionine. Fenugreek saponins support testosterone biosynthesis through modulation of 5α-reductase and stimulation of luteinizing hormone (LH) secretion; black ginger polymethoxyflavones enhance nitric oxide synthesis, mitochondrial activity, and steroidogenic enzyme expression; and zinc methionine, a bioavailable chelated form of zinc, serves as a critical cofactor in testosterone biosynthesis and provides antioxidant protection.^11–18^ The combination offers a multifaceted approach to improving testosterone status, muscle strength, and reproductive health. The formulation was developed under the Natural Ingredients for Mental and Performance (NIMP) Platform, a research and development initiative focused on optimizing the efficacy and delivery of phytoactive compounds for human performance enhancement. Within this framework, InSitu360 molecular complexation technology employed a proprietary encapsulation platform that enables real-time interaction between fenugreek saponins, black ginger polymethoxyflavones, and zinc methionine during processing. This three-stage approach (i) targeted extraction and purification of bioactives, (ii) enrichment of fenugreek saponins, and (iii) *in situ* molecular complexation under controlled shear, pressure, and thermal conditions creates a stable nanostructured matrix. The process enhances solubility, stability, and intestinal absorption while promoting synergistic physiological effects. By improving molecular alignment and sustained release, InSitu360 overcomes common challenges of poor bioavailability in plant-based nutraceuticals and provides a next-generation delivery strategy for testosterone support and muscle endurance, consistent with principles established in spray-drying and nano-encapsulation literature.^19–23^

Specifically, fenugreek-derived steroidal saponins have been reported to influence androgen-associated pathways and sex hormone metabolism, with human studies demonstrating improvements in testosterone-related parameters, strength, body composition, and symptoms associated with androgen deficiency.^24,25^ Black ginger (*Kaempferia parviflora*) polymethoxyflavones have been associated with improvements in mitochondrial bioenergetics, endothelial nitric oxide signaling, physical performance, and male sexual health parameters.^26–28^ Zinc is an essential trace element that serves as a cofactor in numerous enzymatic processes involved in steroidogenesis and reproductive physiology, and zinc deficiency has been consistently associated with reduced testosterone concentrations, while zinc supplementation has been shown to support normal testosterone status in deficient individuals.^29,30^ Accordingly, the formulation strategy was designed to target multiple biological determinants of testosterone status simultaneously rather than relying on a single mechanistic pathway. Given the substantial body of evidence supporting the individual efficacy of these ingredients, our intention was to investigate a novel formulation combining these well-characterized bioactives and to evaluate whether their concurrent administration could provide enhanced benefits compared with existing single-ingredient approaches. The objective of the present study was to formulate and characterize Testolift using InSitu360 technology and to evaluate its physicochemical attributes and efficacy in enhancing serum testosterone levels in male Sprague Dawley rats.

## 2. Materials and methods

### 2.1. Materials

Rhizomes of *Kaempferia parviflora* were sourced from Ava Corporation, Thailand, and seeds of *Trigonella foenum-graecum* (fenugreek) were obtained from Kuber Impex Limited, Indore, India. Botanical authentication of both plant materials was performed by a qualified local botanist, although no voucher specimens were archived. Zinc methionine was procured from Wilincare, China, and phosphatidylcholine was obtained from Shankar Nutricon, India. Gum arabic used in the formulation was purchased from Nexira, France. Reference standards of polymethoxyflavones such as 5,7-dimethoxyflavone (≥95% purity; CAS No. 21392-57-4), 5,7,4′-trimethoxyflavone (≥90% purity; CAS No. 5631-70-9), and 3,5,7,3′,4′-pentamethoxyflavone (≥90% purity; CAS no. 1247-97-8) were purchased from Sigma-Aldrich (India). All organic solvents used in the study, including ultra-performance liquid chromatography (UPLC), were of analytical grade and were procured from Merck India. Ultrapure water generated by a Millipore Milli-Q system was employed throughout the experimental procedures.

### 2.2. Preparation of Testolift using InSitu360 technology

#### 2.2.1. Extraction and purification of methoxyflavones from black ginger

Dried rhizomes of *K. parviflora* (black ginger) were finely powdered to increase the extraction surface area. The dried black ginger rhizome powder (100 g) was subjected to maceration using food-grade ethyl alcohol as the solvent, which effectively solubilizes polymethoxyflavones (PMFs) such as 5,7-dimethoxyflavone (DMF), 5,7,4′-trimethoxyflavone (TMF), and 3,5,7,3′,4′-pentamethoxyflavone (PMF), due to their moderate polarity and lipophilicity. The extraction was performed at ambient temperature to prevent thermal degradation of sensitive flavonoids. The resulting crude extract was filtered and concentrated under reduced pressure to remove the solvent. To further purify the methoxyflavones, the concentrate was subjected to re-extraction with methyl alcohol, which selectively enriches the fraction in methoxyflavones content. The purified extract was analytically standardized to contain not less than 10% total methoxyflavones, comprising multiple PMFs constituents such as DMF, TMF, and PMF, as confirmed by UPLC. After complete solvent removal and drying, 5.2 ± 0.3 g of purified polymethoxyflavone-enriched fraction was obtained from the initial 100 g of dried black ginger powder. The extraction yield was calculated on a dry weight basis using the eqn (1)1
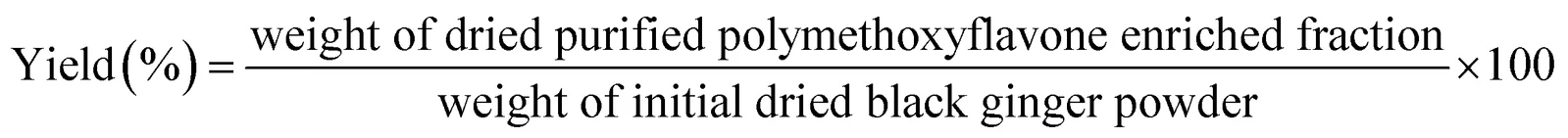


Accordingly, the overall extraction yield of the purified polymethoxyflavone-enriched fraction was 5.2 ± 0.3% (w/w). This multi-component extract retains a broad spectrum of flavonoids known to contribute to the androgenic, metabolic, and antioxidant properties of Testolift.

#### 2.2.2. Extraction and purification of fenugreek saponins

Fenugreek (*T. foenum-graecum*) seeds (100 g) were first mechanically crushed and defatted with hexane to remove lipophilic components, which can interfere with saponin extraction and subsequent formulation stability. The defatted residue was then subjected to hydroalcoholic extraction using a 70 : 30 (v/v) ethanol : water mixture, at a raw material-to-solvent ratio of 1 : 5. This solvent system efficiently extracts polar saponins and glycosides while minimizing the extraction of proteins and polysaccharides. The extraction was performed in five sequential cycles, each involving soaking for 1 hour, to maximize saponin yield. The combined extracts (miscella) were concentrated under reduced pressure. The concentrated extract was dissolved in methanol and sequentially washed to remove soluble sugars and polar impurities. The washed methanol fractions were concentrated, and the residue was partitioned with ethyl acetate to remove non-saponin organic components. This purification workflow yielded a multi-component saponin-rich extract containing 72% total saponins, predominantly furostanol-type glycosides. After complete solvent removal and drying, 6.5 ± 0.4 g of purified saponin-enriched fraction was obtained from the initial 100 g of dried fenugreek seed material. The extraction yield was calculated on a dry weight basis using the eqn (2)2
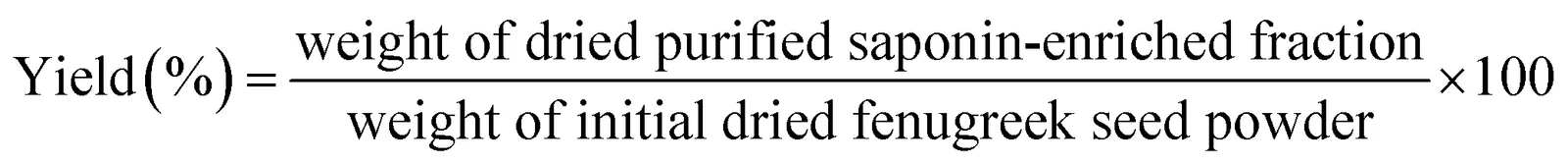


Accordingly, the overall extraction yield of the purified fenugreek saponin-enriched fraction was 6.5 ± 0.4% (w/w). This multi-step enrichment process minimizes non-active matrix components and enhances the bioactive density and structural stability of the fenugreek fraction used in Testolift.

#### 2.2.3. Preparation of Testolift composite using InSitu360 technology

Testolift was prepared under the NIMP Platform using a proprietary InSitu360 molecular complexation process. Accurately weighed portions of saponin-enriched (80% saponins) fenugreek extract (25 g) and black ginger extract (25 g) were dissolved in hot distilled water (50 °C) to enable solubilization and partial hydration of bioactives. The mixture was homogenized using an IKA T18 Digital Ultra-Turrax Homogenizer (Germany) at 12 000 ± 50 rpm for 10 min to reduce particle size and increase the surface area for molecular interactions. The homogenized slurry was further processed through high-pressure homogenization at 500 bar for three successive cycles to achieve uniform nanoscale dispersion and promote molecular interaction between saponins, flavonoids, and matrix components. Subsequently, 8 g zinc methionine was incorporated and homogenized to facilitate coordination bonding with saponins and flavonoids, improving solubility and stability. Gum arabic (35 g) and lecithin (7 g) were then added as stabilizing and encapsulating agents, enhancing emulsification, lamellar structure formation, and overall bioactive protection. The final mixture underwent an additional high-shear and high-pressure homogenization step to ensure uniform dispersion. The homogeneous slurry was then spray-dried using a Spray Tech Systems Mini Laboratory Spray Drier (STS001-SS) under controlled conditions: inlet temperature 175–185 °C, outlet temperature 75–85 °C, and drying air pressure 10 kg cm^−2^. Spray drying enabled rapid water removal and encapsulation of bioactives within a protective gum arabic–lecithin matrix, conferring enhanced stability, dispersibility, and shelf life, in line with previously reported microencapsulation approaches for bioactives.^20–22^ A schematic overview of the InSitu360-based Testolift formulation process is presented in Fig. 1.

**Fig. 1:**
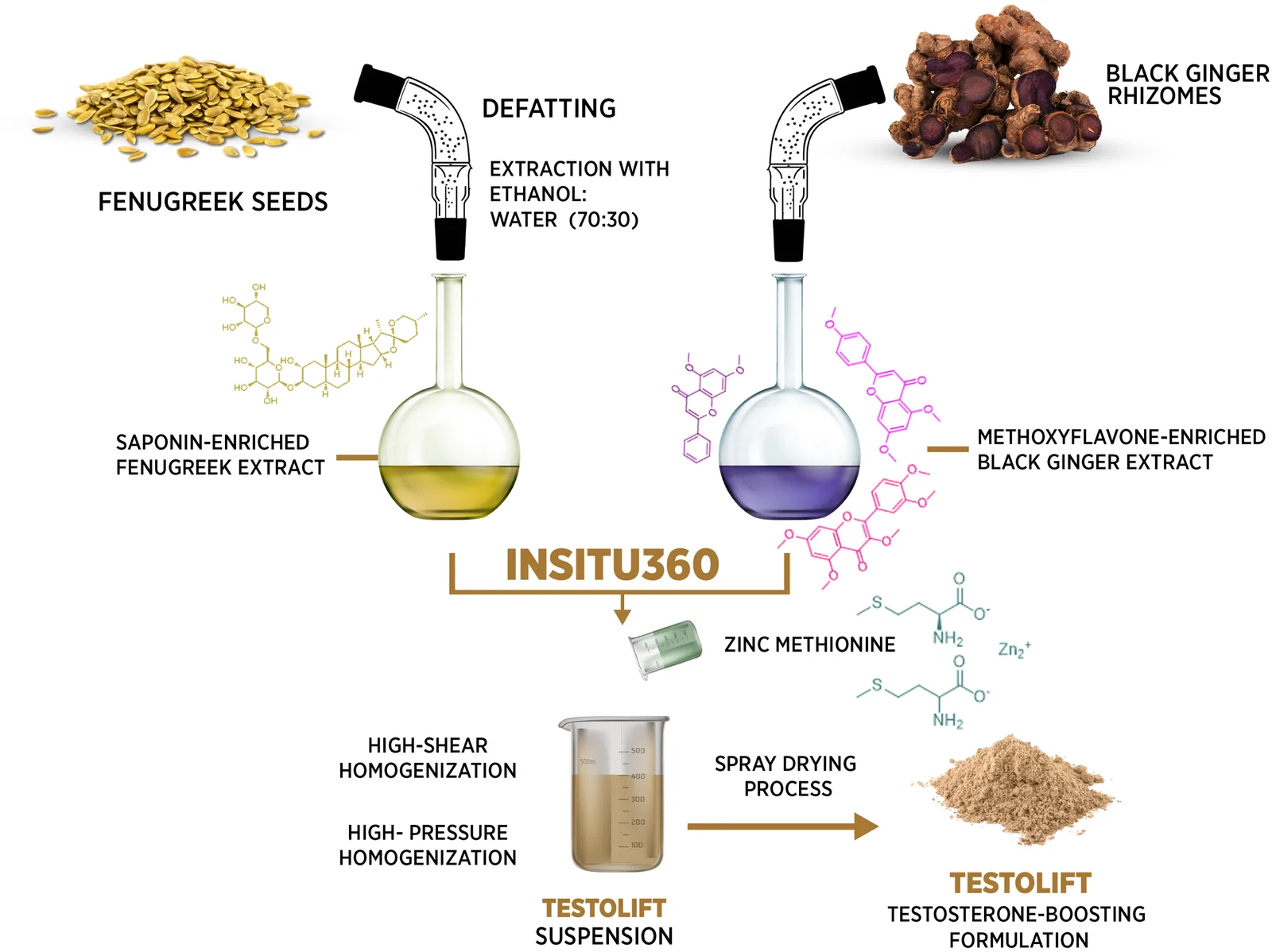
Schematic overview of InSitu360 molecular complexation showing extraction, enrichment, homogenization, and spray-drying steps in developing the Testolift: testosterone-boosting nutraceutical formulation.

### 2.3. Analysis of Testolift

#### 2.3.1. Quantification of polymethoxyflavones in Testolift by ultra-performance liquid chromatography (UPLC)

The quantification of major PMFs in Testolift including DMF, TMF, and PMF was performed using a Shimadzu Prominence P-Series HPLC system equipped with a quaternary gradient pump, autosampler, and photodiode array (PDA) detector, controlled through LabSolutions software (Shimadzu Corp., Japan). Reference standards of DMF, TMF, and PMF (≥90–95% purity, Sigma-Aldrich) were weighed accurately, dissolved in methanol, and combined to prepare a mixed-stock solution (1000 mg L^−1^). Working mixed standards were freshly prepared at 10, 20, 40, 60 and 80 mg L^−1^, filtered through a 0.2 µm nylon membrane, and injected for constructing calibration curves.

For sample preparation, 50 mg of Testolift powder was transferred into a 25 mL volumetric flask and extracted with 20 mL of methanol under sonication for 30 min. The mixture was brought to volume with methanol, vortexed, and filtered through a 0.2 µm nylon membrane. The resulting filtrate was analyzed directly, with further dilution performed when necessary to ensure the analyte peak area fell within the validated calibration range.

Chromatographic separation was achieved using a mobile phase of methanol and 1% acetic acid (60 : 40, v/v) at a flow rate of 1.0 mL min^−1^, with a 20 µL injection volume. The PDA detector was set at 260 nm. The mobile phase was degassed and filtered (0.45 µm) prior to use. The analytical procedure was validated according to ICH Q2(R1) guidelines. System suitability parameters, including retention time, theoretical plate count, USP tailing factor, chromatographic resolution between adjacent peaks, and PDA-based peak purity, were evaluated in accordance with ICH Q2(R1); detailed results are provided in the SI (Fig. S1 and Table S1).

Calibration curves for DMF, TMF, and PMF demonstrated excellent linearity across 10–80 mg L^−1^, with correlation coefficients (*R*^2^) ≥ 0.999. Repeatability was confirmed with a % RSD of 1.171% (Table S2), and accuracy ranged from 86.74% to 100.05% recovery (Table S3), supporting the robustness of the method. The precision study across concentrations 10–60 mg L^−1^ showed % RSD values ranging from 0.08% to 1.19% (Table S4). The method demonstrated high sensitivity, with a limit of detection (LOD) of 5 mg L^−1^ (Fig. S11) and a limit of quantification (LOQ) of 10 mg L^−1^ (Fig. S12). All validated results confirmed the sensitivity and robustness of the analytical procedure for PMF determination in the Testolift matrix. Representative chromatograms of the mixed PMF standards at 10–80 mg L^−1^ and the calibration curve are provided in Fig. S2–S7. Chromatograms of the black-ginger extract, Testolift formulation, and blank solvent are presented in Fig. S8–S10. All analytes exhibited consistent retention times in both standards and sample chromatograms (RT ≈ 8.9, 11.4, and 13.2 min for PMF, DMF, and TMF respectively), with no interfering peaks and acceptable symmetry and tailing factors. These results confirm the specificity, selectivity, and suitability of the validated UPLC method for quantifying PMFs in Testolift.

#### 2.3.2. Estimated total saponin content in Testolift

The quantification of total saponin content was carried out using a gravimetric extraction and precipitation method based on successive solvent extraction, selective precipitation, and drying to constant weight. This technique enables quantitative recovery of saponins while eliminating nonpolar impurities through selective solvent partitioning. The approach remains widely used and validated for saponin-rich extracts, including fenugreek and similar medicinal plants, owing to its reproducibility and simplicity for complex matrices. Recent reports confirm that gravimetric and chromatographic methods yield comparable quantitative accuracy when validated for linearity, precision, and recovery in botanical extracts.^31,32^ Approximately 5 g of the Testolift was refluxed with 25 mL of 90% v/v methanol for 30 minutes. The residue was further extracted twice with an additional 25 mL methanol under the same conditions. All methanolic extracts were pooled and the solvent was distilled off to yield a semi-solid ‘soft extract’. The soft extract was treated successively with 25 mL petroleum ether (refluxed for 30 minutes), then cooled and decanted. This was followed by sequential treatments with 25 mL chloroform and 25 mL ethyl acetate to remove interfering non-polar components. The residual soft extract was redissolved in 25 mL of 90% v/v methanol, filtered, and concentrated to 5 mL. The concentrated methanol extract was added dropwise, with constant stirring, to 25 mL of acetone to precipitate the saponins. The resulting precipitate was collected by filtration and dried to a constant weight in a hot air oven at 105 °C. Saponin percentage was calculated based on the dry weight of the precipitate (*W*_2_) relative to the initial sample weight (*W*_1_) using the following eqn (3)3
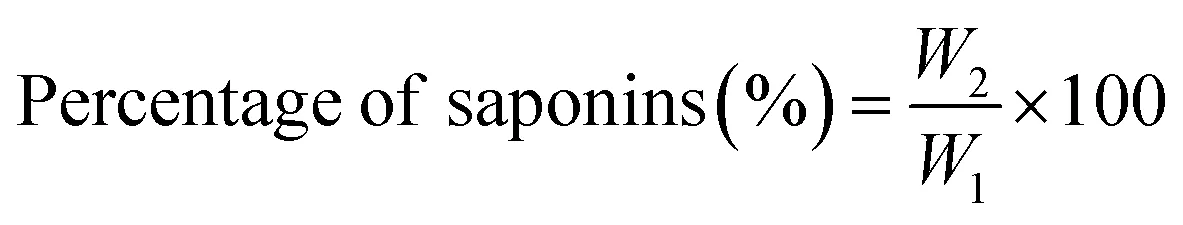


#### 2.3.3. Determination of zinc content in the Tesolift by inductively coupled plasma mass spectrometry (ICP-MS)

Zinc content in the formulation was determined using Inductively Coupled Plasma Mass Spectrometry (ICP-MS) equipped with an argon plasma source and a mass analyzer. Method validation included linearity, recovery, and precision assessments following ICH Q2(R1) guidelines. Testolift (0.5 g) was accurately weighed into a microwave digestion vessel. To this, 5.0 mL of concentrated nitric acid, 0.5 mL of hydrochloric acid, and 1.0 mL of hydrogen peroxide were added. After allowing the mixture to self-digest for 15 minutes, the vessels were sealed and subjected to microwave-assisted digestion. The digest was then transferred quantitatively into a 50 mL volumetric tube and diluted to volume with ultrapure water. The digested samples were introduced into the ICP-MS, and the zinc content was quantified.

### 2.4. Physicochemical characterization

#### 2.4.1. Determination of color attributes (L*, a*, b* values)

The determination of color attributes (L*, a*, b*) of the samples such as black ginger extract, fenugreek seed extract and Testolift was conducted using a color spectrophotometer (Sensegood instrument) based on CIELAB color space coordinates. The sample was uniformly spread on a transparent plastic plate. The instrument was set to spectrum mode using the first icon (graph symbol) to display the full color spectrum. Once stabilized, the ‘gridline’ option (second icon) was selected to obtain direct readings of L* (lightness), a* (red-green), and b* (yellow–blue) values. All measurements were conducted in triplicate and mean values were recorded.

#### 2.4.2. Color measurements by UV direct method

Accurately 1 g of the test samples were weighed and transferred to a 100 mL volumetric flask. The volume was made up to the mark with demineralized water, ensuring complete dissolution. The absorbance of the resulting solution was measured at 628 nm using the UV-visible spectrophotometer (UV-1900i UV-vis spectrophotometer, Japan). For stability assessment, the absorbance of the same sample was re-measured at 628 nm after 16 hours under ambient laboratory conditions. This allowed evaluation of color change or pigment precipitation.

#### 2.4.3. Bulk density and tapped density

The bulk and tapped densities of the powdered sample were determined in accordance with USP 〈616〉.^33^ This method is widely employed in pharmaceutical and nutraceutical quality control to evaluate the compressibility, flowability, and packaging behavior of powders. A clean, dry 250 mL plastic graduated cylinder was first placed on a calibrated analytical balance and tared. Approximately 200 g of the test powder was slowly introduced into the cylinder through a powder funnel, taking care to avoid any compression. The funnel was removed, and the weight of the sample was recorded (*W*, in grams), and the initial volume occupied by the sample (*V*_1_, in mL) was noted as the bulk volume. The cylinder was then tapped 60 times from a consistent drop height to allow powder consolidation. After tapping, the final volume (*V*_2_, in mL) was recorded as the tapped volume. Bulk density was calculated as eqn (4) and tapped density was calculated as eqn (5).4
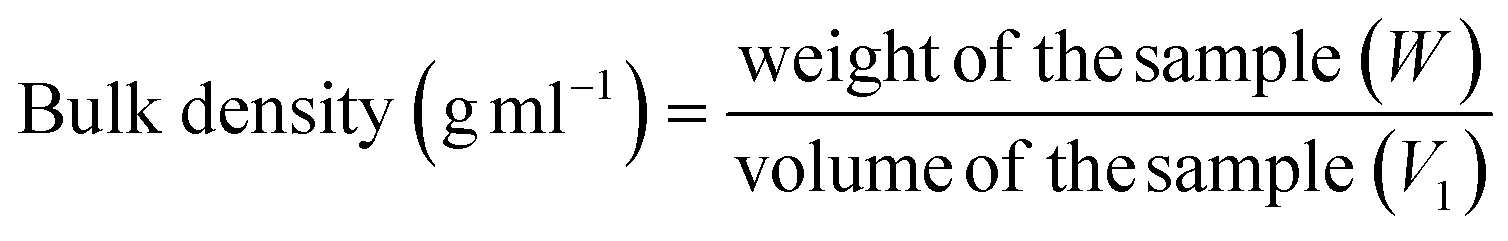
5
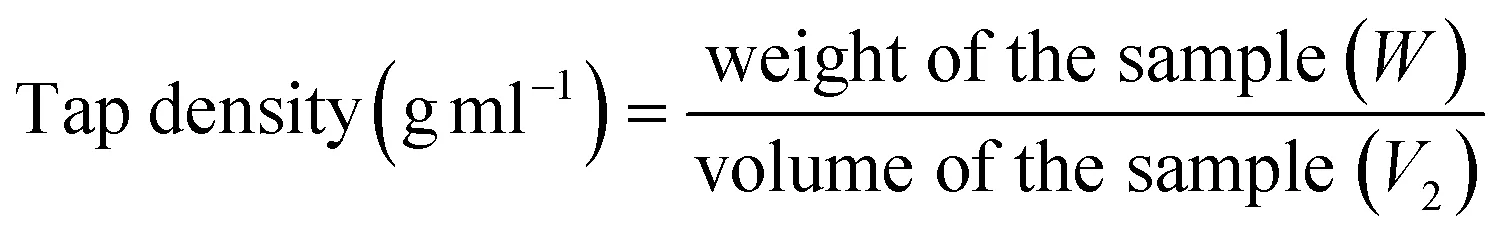


#### 2.4.4. Water solubility index (WSI) and moisture content

The water solubility index (WSI) of black ginger extract, fenugreek seed extract and Testolift was determined following the method described by Amalraj *et al.*^34^ Briefly, 2.5 g of each sample was mixed with 30 mL of distilled water in a 100 mL centrifuge tube and vigorously shaken. The mixture was then incubated in a water bath at 37 °C for 30 min. followed by centrifugation at 10 000 rpm for 30 min. The resulting supernatant was carefully transferred to pre-weighted Petri dishes and dried in an oven at 103 ± 2 °C. The WSI (%) was calculated as the percentage of the dried supernatant relative to the initial 2.5 g of sample.

Moisture content was determined by the Loss on Drying (LOD) method. Prior to use, LOD bottles were pre-dried in a hot air oven at 105 °C for 60 min and then allowed to cool in a desiccator at room temperature for 30 min. Subsequently, 1 g of each sample was accurately weighted into the pre-conditioned LOD bottles. The samples were then dried in the over at 105 °C for 5 h. After drying, the bottles were transferred to the desiccator and cooled for 30 min at room temperature before reweighing. The percentage of moisture content was calculated using eqn (6), based on the weight loss before and after drying.6
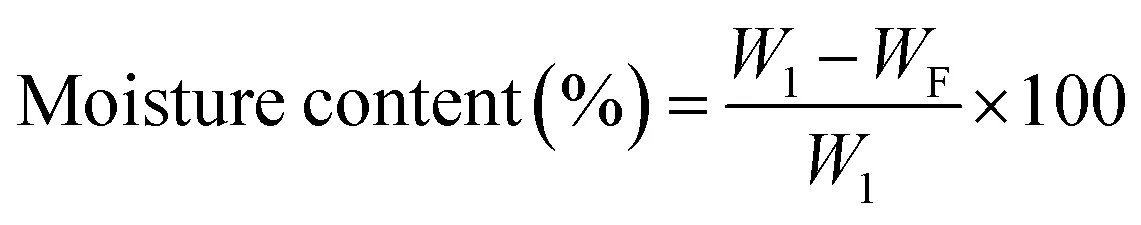
where *W*_1_ is the initial weight of the sample and *W*_F_ is the final weight of the sample.

#### 2.4.5. Hygroscopicity

The hygroscopic behavior of black ginger extract, fenugreek seed extract and Testolift was evaluated by uniformly distributing 1 g of each powder sample across Perti dishes to maximize the surface area exposed to ambient humidity. The dishes were placed in a desiccator maintained at 23 °C and 76% relative humidity, the latter controlled using a nitric acid solution. After 90 minutes of exposure, the samples exhibited a measurable increase in mass due to moisture absorption. Although hygroscopicity is typically characterized by equilibrium moisture content, a comparative analysis was conducted by quantifying the moisture uptake per gram of drying powder following the 90 minutes exposure period at 76% relative humidity.^34^

#### 2.4.6. Degree of caking (DC)

To evaluate the degree of caking, powder samples were first dried in a hot air oven at 70 °C and then allowed to cool to room temperature. The dried samples were accurately weighted and subsequently transferred to a 420 mm sieve. The sieving was performed for 5 min using a mechanical shaker to ensure uniform agitation. Following sieving, the mass of powder retained on the sieve was recorded. The caking index was then calculated using eqn (7), based on the proportion of material retained relative to the total initial sample weight.7
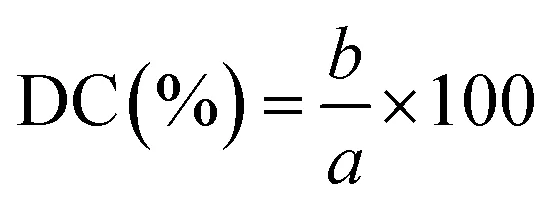
where DC represents the degree of caking (%), “*a*” is the initial amount of powder used in the sieving process, and “*b*” is the amount of powder remaining on the sieve after sieving.^35^

All physicochemical measurements were performed in triplicate (*n* = 3), and results are expressed as mean ± standard deviation (SD).

#### 2.4.7. Fourier transform infrared spectroscopy (FTIR)

The FTIR spectra of the individual components (fenugreek extract, black ginger extract, and zinc methionine) and the final formulation Testolift were recorded using a Fourier Transform Infrared Spectrometer (Thermo Scientific Nicolet iS5 FT-IR Spectrometer, USA) equipped with an attenuated total reflectance (ATR) accessory. Samples were scanned over the range of 4000–500 cm^−1^ at a resolution of 4 cm^−1^ with 32 cumulative scans. Characteristic absorption bands were analyzed to confirm the presence of key functional groups and to detect any shifts or changes indicative of molecular interactions in the composite formulation.

#### 2.4.8. Field emission scanning electron microscopy (FE-SEM) with energy dispersive X-ray spectroscopy (EDS) analysis

The surface morphology and microstructure of Testolift were examined using Field Emission Scanning Electron Microscopy (FE-SEM, ZEISS, Germany). Samples were mounted on aluminum stubs using double-sided carbon tape and sputter-coated with a thin layer of gold to enhance conductivity. Micrographs were captured at various magnifications (100×, 1000×, 2500×, and 5000×) under an accelerating voltage of 3.0 kV. The images were analyzed for particle size, shape, surface texture, and dispersion of the constituent bioactives within the composite matrix.

EDS analysis was conducted to determine the qualitative and semi-quantitative elemental composition of the Testolift. Point detection and area mapping modes were used to ensure broad surface characterization. Elemental mapping was carried out with a dwell time of 50 microseconds per pixel across a scanning area of approximately 100 µm^2^. Elemental profiles were obtained for carbon (C), oxygen (O), nitrogen (N), sulfur (S), potassium (K), and zinc (Zn).

#### 2.4.9. Transmission electron microscopy (TEM)

The ultrastructural morphology and particle size distribution of Testolift were further investigated by Transmission Electron Microscopy (TEM, JEOL, Japan). A small amount of Testolift was dispersed in ethanol and sonicated for 10 minutes to ensure uniform suspension. A drop of the suspension was placed on a carbon-coated copper grid and allowed to air-dry. The grids were then examined at accelerating voltages of 80–120 kV. Images were obtained at magnifications ranging from 50 nm to 1 µm, allowing visualization of the internal structure, particle shape, and encapsulation characteristics at the nanoscale.

#### 2.4.10. Particle size distribution and zeta potential analysis

The particle size distribution and zeta potential of Testolift were determined by dynamic light scattering (DLS) using a Malvern Zetasizer Nano ZS (Malvern Instruments, UK) at 25 °C. Samples were appropriately diluted with deionized water to avoid multiple scattering effects. The hydrodynamic diameter was calculated based on the Stokes–Einstein equation, and zeta potential values were obtained from electrophoretic mobility measurements using Nano DTS software (version 6.34). All measurements were performed in triplicate, and results are presented as mean values.

#### 2.4.11. Differential scanning calorimetry (DSC)

Thermal behavior and physical interactions among the bioactives were assessed using differential scanning calorimetry (DSC, TA Instruments, USA). Approximately 5–10 mg of each sample (black ginger extract, fenugreek seed extract, zinc methionine, and Testolift composite) was accurately weighed and sealed in an aluminum pan. The samples were scanned from 30 °C to 350 °C at a heating rate of 10 °C min^−1^ under a constant nitrogen flow of 50 mL min^−1^. Thermograms were recorded, and endothermic/exothermic transitions were analyzed to determine melting points, decomposition temperatures, and evidence of molecular-level interactions or changes in crystallinity within the composite formulation.

#### 2.4.12. Storage stability study of Testolift

A detailed storage stability study was conducted to evaluate both the chemical and physicochemical stability of the Testolift formulation over a period of 180 days. Testolift is intended to be stored at room temperature, and the stability study was performed in accordance with the principles outlined in the ICH Q1A(R2) guideline. The formulations were stored under the following conditions 25 ± 2 °C/60% RH ± 5% (ambient/long-term storage condition), 45 ± 2 °C/75% RH ± 5% (elevated-temperature stress condition) and 4 ± 2 °C (supplementary low-temperature storage condition; RH not actively controlled). The refrigerated condition was included as a supplementary condition to evaluate formulation behavior under low-temperature storage. Samples were stored in closed polyethylene zip-lock pouches sealed within aluminum laminate pouches, providing moisture barrier protection consistent with nutraceutical raw material packaging practices.

Samples were collected at predetermined intervals (Day 0, 30, 60, 90, 120, 150, and 180) and analyzed in triplicate (*n* = 3). Chemical stability was assessed through quantification of the key bioactive constituents methoxyflavones, saponins, and zinc. Methoxyflavones were quantified using the validated UPLC method, saponins were determined *via* the established gravimetric precipitation method, and zinc content was analyzed using ICP-OES described in Section 2.3. The stability of each constituent was expressed as the percentage of its initial concentration, calculated according to eqn (8):8
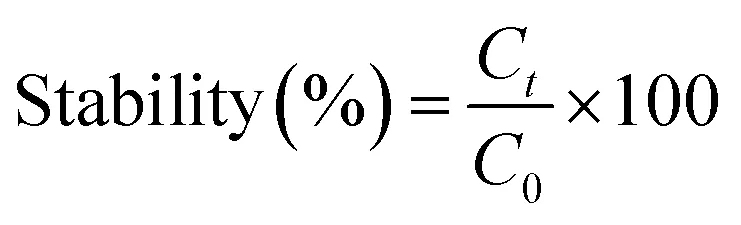
where *C*_0_ is the initial concentration (at day 0) and *C*_*t*_ represents the concentration at the specified time point *t*. The data were expressed as mean ± standard deviation (SD) from three independent determinations.

Physicochemical stability was also assessed concurrently by monitoring changes in color (L*, a*, b* values), bulk density, tapped density, moisture content, hygroscopicity, water solubility index (WSI), and degree of caking described in Section 2.4. These parameters were selected because they are sensitive indicators of powder integrity, moisture uptake, flow characteristics, and potential degradation in spray-dried botanical formulations. This combined assessment allowed for a comprehensive evaluation of both compositional and functional stability, consistent with established practices for microencapsulated and nano-structured nutraceutical ingredients. All chemical and physicochemical stability evaluations were performed in triplicate (*n* = 3) at each time point, and data are expressed as mean ± SD.

#### 2.4.13. Microbiological quality evaluation of Testolift

Microbiological evaluation of the Testolift formulation was carried out to ensure compliance with safety requirements for herbal and nutraceutical products. The testing included determination of total aerobic plate count (TAPC), total yeast and mold count (TYMC), coliforms, *Escherichia coli*, *Salmonella* spp. and *Staphylococcus aureus*. All analyses were performed according to ISO standard methods. The aerobic plate count was determined as per ISO 4833-1, coliforms by ISO 4832, *Escherichia coli* by ISO 16649-2, *Salmonella* spp. by ISO 6579-1, *Staphylococcus aureus* by ISO 6888-1:2021, and yeasts and molds by ISO 21527-2:2008. Results were expressed as colony-forming units per gram (CFU per g), and the limit of quantification (LOQ) for all enumerated microorganisms was 10 CFU per g. Detection of *Salmonella* was reported as presence or absence per 25 g of the sample. All analyses were conducted on samples in good condition and tested under aseptic laboratory conditions.

### 2.5. Study design and experimental procedures for efficacy evaluation of Testolift on serum testosterone in male rats

#### 2.5.1. Study design and ethical compliance

This *in vivo* experimental study was conducted to evaluate the effect of Testolift on serum testosterone levels in male Sprague Dawley rats. All experimental procedures were performed in strict accordance with the guidelines of the Committee for the Control and Supervision of Experiments on Animals (CCSEA), Government of India (Registration No. 1803/PO/RcBi/S/2015/CCSEA). The protocol was reviewed and approved by the Institutional Animal Ethics Committee (IAEC) prior to study initiation. All *in vivo* activities complied with the ethical standards for laboratory animal care, including housing, handling, dosing, and euthanasia. Furthermore, the analytical measurements performed for testosterone, methoxyflavones, saponins, and zinc adhered to the International Council for Harmonisation (ICH) Q2(R1) guidelines for method validation, ensuring accuracy, precision, linearity, and reproducibility of the analytical outcomes.^36^

#### 2.5.2. Experimental animals

Male Sprague Dawley rats, aged approximately 10 weeks and weighing 250–280 g, were procured from an accredited breeder. Animals were housed in polypropylene cages in a controlled environment: temperature 22 ± 3 °C, relative humidity 30–70%, and a 12 hours light/dark cycle. Animals were individually marked and assigned to groups using randomization based on body weight, ensuring that the variation did not exceed ±20% of the mean body weight. Each group was housed separately and identified by cage numbers. Cages were cleaned daily with disinfectants. All animals received standard chow diet and reverse osmosis (RO) water ad libitum. Water was routinely monitored for microbial contamination, and bottles were regularly inspected for proper operation. Sixteen animals were randomized into two groups (*n* = 8 per group): group I (Normal Control): 0.5% carboxymethylcellulose (CMC) orally and Group II (Testolift): 30 ± 0.5 mg kg^−1^ body weight of Testolift in 0.5% CMC. The formulation was administered once daily by oral gavage at approximately the same time each day (±1 hour) for 42 consecutive days.

#### 2.5.3. Observations and sample collection and analysis

Animals were monitored at least twice daily for mortality, moribund state, or abnormal behavior. Cage-side evaluations for visible clinical signs were conducted daily. Detailed clinical examinations were performed prior to treatment and weekly thereafter, assessing skin, fur, eyes, mucous membranes, and behavioral patterns. Individual body weights were recorded at receipt, on the day of randomization, on Day 1 (pre-dose), and weekly thereafter. Feed consumption was monitored and recorded weekly. Blood samples were collected from all animals on Day 0 (baseline) and Day 42 (end of study). Animals were fasted overnight prior to sample collection. Under isoflurane anesthesia, approximately 1 mL of blood was withdrawn *via* the retro-orbital route. Blood samples were centrifuged at 10 000 rpm for 10 minutes to separate serum, which was then stored at −80 °C until analysis. Serum testosterone levels were quantified using a fully automated clinical chemistry analyzer (MISPA ace, Agape Bio-medicals Ltd).

#### 2.5.4. Statistical analysis

All data, including body weight, feed consumption, and biochemical parameters, were analyzed using GraphPad Prism software (version 5.01). Results are presented as mean ± standard deviation (SD). Statistical comparisons between control and treated groups were performed using *t*-tests, with significance set at *p* < 0.05.

## 3. Results and discussion

### 3.1. Advanced botanical formulation *via* InSitu360 molecular complexation technology: a breakthrough in synergistic delivery

The Testolift formulation was developed using the proprietary InSitu360 Molecular Complexation Technology, designed to achieve real-time molecular interactions between*Kaempferia parviflora* (black ginger) extract, *Trigonella foenum-graecum* (fenugreek) saponin-enriched extract, and zinc methionine. This process facilitates molecular interaction through controlled pH, temperature, and solvent polarity, producing a homogeneous nano-dispersion with enhanced physicochemical stability and bioactive retention. The resulting composite exhibited uniform particle distribution (sub-200 nm), improved aqueous dispersibility, and high encapsulation efficiency, as confirmed by FT-IR, DSC, and morphological analyses. The integration of methoxyflavones, furostanol-type saponins, and zinc methionine was evidenced by characteristic spectral shifts and the absence of individual component peaks, indicating strong molecular interactions and complex formation. This synergistic matrix architecture promotes higher solubility and stability of plant actives, potentially enhancing their intestinal permeability and systemic bioavailability. The formulation design thus supports multi-targeted efficacy, combining the adaptogenic and ergogenic actions of methoxyflavones, the anabolic potential of saponins, and the enzymatic cofactor role of zinc. The optimized Testolift complex demonstrates a stable and reproducible system suitable for diverse nutraceutical delivery formats.

### 3.2. Quantitative analysis of bioactive components

The validated analytical methods confirmed robust quantities of the targeted bioactives within Testolift. The formulation contained 0.92% PMFs (by UPLC), 20% total saponins (by gravimetric precipitation), and 1.4% elemental zinc (by ICP-MS). These quantitative results underline that Testolift is a high-potency nutraceutical formulation with well-balanced bioactive loading designed to support testosterone enhancement. Using mixed reference standards of DMF, TMF, and PMF, the UPLC method exhibited excellent linearity across 10–80 mg L^−1^, with correlation coefficients (*R*^2^ ≥ 0.999) for all three polymethoxyflavones (Fig. S7). The method exhibited high analytical accuracy, with mean spike recoveries ranging from 86.74% to 100.05% (Table S3). Precision was confirmed through intra-day repeatability (%RSD = 1.171%, Table S2), while precision evaluated across multiple concentration levels (10–60 mg L^−1^) yielded % RSD values ranging from 0.08% to 1.19% (Table S4). All % RSD values were within the generally accepted ≤2% criterion for chromatographic precision, confirming the suitability of the method for routine quantitative analysis. Collectively, these parameters confirm that the method is robust, accurate, and reliable for routine quantitative assessment of PMFs in complex nutraceutical matrices.

Fig. 2 presents representative UPLC chromatograms of (a) mixed PMF standards at 20 mg L^−1^, (b) *Kaempferia parviflora* extract, and (c) the Testolift formulation. The chromatograms showed well-resolved and symmetrical peaks with consistent retention times of approximately 8.8–9.0 min (PMF), 11.2–11.5 min (DMF), and 12.9–13.3 min (TMF) across standards, extract, and formulation samples. For analytical transparency, chromatograms of PMF standards across the calibration range (10–80 mg L^−1^), calibration curves, blank solvent, precision, accuracy, LOD, and LOQ assessments are provided in the SI (Fig. S1–S12). No baseline instability, co-eluting peaks, or matrix interferences were observed, confirming the specificity and selectivity of the analytical method. Importantly, the chromatographic profile of Testolift closely matched that of the mixed standards, confirming the successful incorporation and chemical integrity of DMF, TMF, and PMF within the formulation. The improved peak symmetry and clarity observed in the updated SI reflect the optimized method performance following the use of lower-concentration standards and matrix-matched dilutions. These findings further indicate that the InSitu360 process effectively retained and uniformly dispersed lipophilic methoxyflavones within the gum arabic–lecithin matrix, preventing aggregation or degradation during processing. Literature reports indicate that polymethoxyflavones are associated with enhanced mitochondrial function, cAMP signaling, and nitric oxide-mediated pathways, which are mechanistically linked to improved cellular energy metabolism.^37–39^

**Fig. 2:**
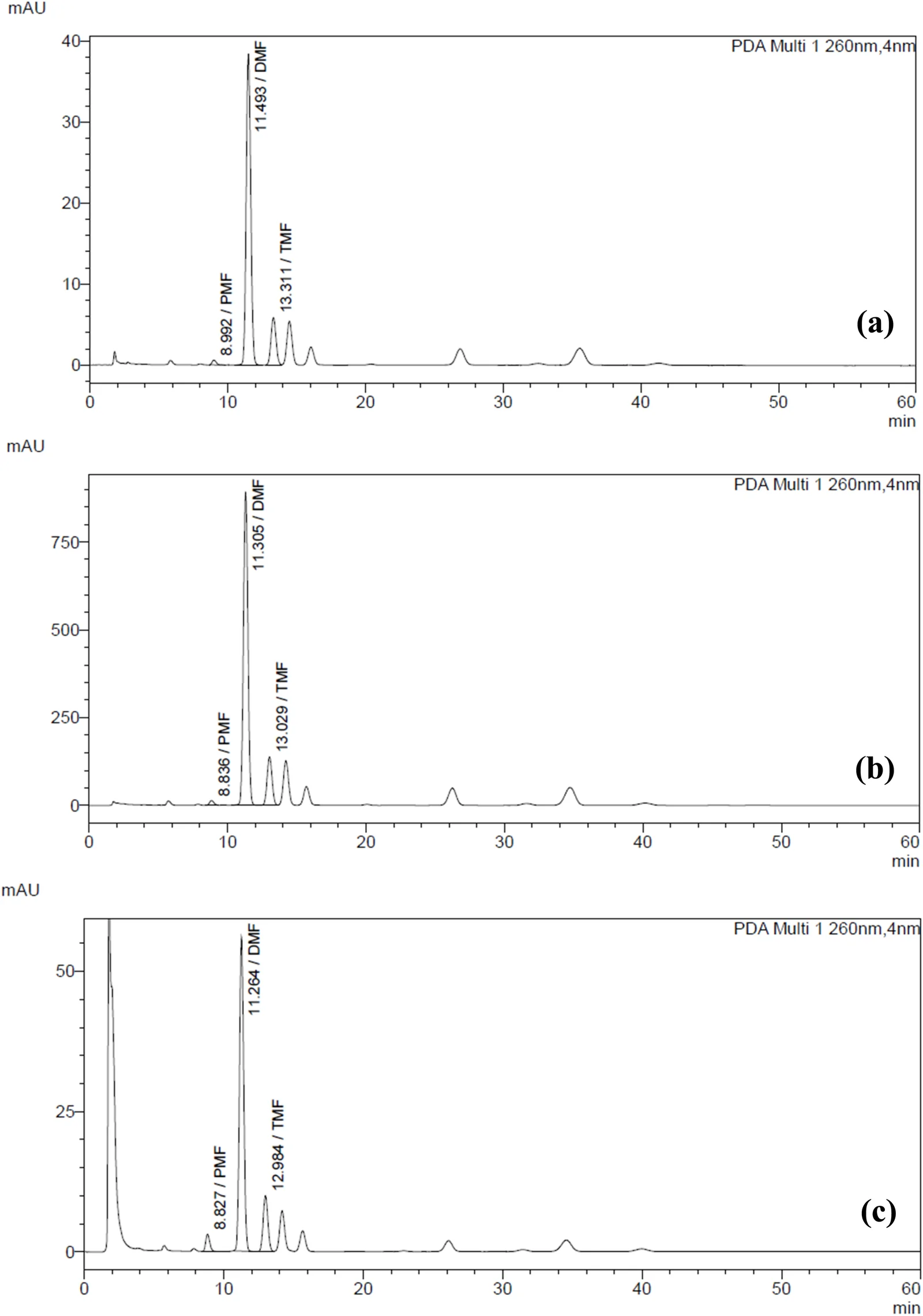
Representative UPLC chromatograms showing (a) mixed polymethoxyflavone (PMF) reference standards at 20 mg L^−1^ comprising DMF, TMF, and PMF; (b) *Kaempferia parviflora* extract; and (c) the Testolift formulation.

The total saponin content (20% w/w), measured by a validated gravimetric method following AOAC and WHO guidelines, confirms the enrichment of steroidal saponins derived from fenugreek. This high concentration supports the functional role of saponins in modulating hormonal balance by stimulating luteinizing hormone (LH) release and suppressing 5α-reductase and aromatase activity, which enhances testosterone biosynthesis.^31,32^ The results also suggest strong retention and compatibility of saponins within the InSitu360 complex, contributing to overall formulation stability. Zinc quantification by ICP-MS revealed 1.4% w/w elemental zinc, in agreement with the theoretical composition of zinc methionine. The validated ICP-MS method demonstrated excellent linearity (*R*^2^ > 0.999), precision (RSD <2%), and recovery (98–102%), confirming analytical robustness. Zinc is an essential cofactor for testicular steroidogenic enzymes such as 3β-HSD and 17β-HSD, contributing to testosterone synthesis, spermatogenesis, and antioxidant protection.^40,41^ Collectively, these results confirm that the InSitu360 process preserved the structural and functional integrity of the active compounds. The uniform distribution of PMFs, saponins, and zinc within the Testolift matrix supports its synergistic biological potential mediated by endocrine modulation, enhanced nutrient bioavailability, and improved physicochemical stability.

### 3.3. Physicochemical characterization

The physicochemical properties of the individual extracts (black ginger and fenugreek) and the final Testolift formulation were assessed and compared (Table 1). During extraction, the black ginger extract appeared as a thin to moderately viscous, sticky paste with a characteristic aromatic odor, while the fenugreek extract was a brown to dark brown, thick, viscous, semi-solid with a bitter odor. Both extracts were subsequently dried to obtain free-flowing powders for evaluation. The final Testolift composite, produced through the InSitu360 process, appeared as a light beige, fine, free-flowing powder with improved dispersibility and low hygroscopicity due to encapsulation within the gum arabic–lecithin matrix. Colorimetric analysis using L*, a*, b* coordinates showed substantial visual differences between raw materials and the final Testolift. The L* value, which indicates lightness, was highest in Testolift (69.05 ± 0.08), suggesting an overall brighter appearance, followed by fenugreek (56.94 ± 0.04) and black ginger (34.61 ± 0.09). The a* and b* coordinates confirmed a redder and more yellow tone in fenugreek, whereas black ginger exhibited a duller hue. The intermediate and more neutral color of Testolift suggests effective blending and molecular integration of the bioactives, masking the intense hues of the individual extracts. UV-visible color stability data showed minimal absorbance change in raw extracts after 16 hours, while Testolift maintained high and consistent absorbance (3.451 and 3.44), reflecting better pigment retention and matrix stabilization. The bulk and tapped density values of Testolift (0.37 and 0.52 g mL^−1^) were lower than those of the raw extracts, suggesting improved compressibility and packing behavior, useful in encapsulation or tablet formulation. Fenugreek showed the highest tapped density (0.92 g mL^−1^), indicative of its dense structure. Water solubility index (WSI) was highest for fenugreek (8.84%), followed by Testolift (6.85%) and black ginger (5.96%). The moderate WSI in Testolift suggests good dispersibility, owing to nano-processing and improved surface area. Moisture content in Testolift (3.56%) was slightly higher than in black ginger and fenugreek, which may be attributed to increased hygroscopic matrix components and residual process moisture. Despite this, its hygroscopicity was lowest (0.09%) compared to black ginger (1.41%) and fenugreek (1.02%), suggesting that Testolift is less prone to moisture uptake, which can improve shelf stability. Degree of caking was relatively higher in Testolift (1.72%), likely due to the interaction between hydrophilic components and fine particle size. Overall, these results demonstrate that Testolift exhibits superior flowability, dispersibility, and physicochemical stability compared to the parent extracts, confirming successful matrix integration and complex encapsulation achieved through InSitu360 technology.

**Table 1:** Physicochemical properties of black ginger extract, fenugreek extract, and Testolift (mean ± SD, *n* = 3)

Parameters	Black ginger extract	Fenugreek extract	Testolift
Color measurement (L*, a*, *b* values)	34.61 ± 0.09, 10.95 ± 0.43, 16.77 ± 2.57	56.94 ± 0.04, 12.96 ± 0.02, 40.77 ± 0.05	69.05 ± 0.08, 7.60 ± 0.21, 23.32 ± 2.25
Colour measurements (by UV-direct)	0.318	0.025	3.451
Colour measurements (by UV-after 16 h)	0.316	0.025	3.44
Bulk density (g mL^−1^)	0.40 ± 0.02	0.55 ± 0.04	0.37 ± 0.03
Tapped density (g mL^−1^)	0.61 ± 0.03	0.92 ± 0.05	0.52 ± 0.02
Water solubility index (%)	5.96 ± 0.07	8.84 ± 0.12	6.85 ± 0.09
Moisture content (%)	0.63 ± 0.04	2.84 ± 0.07	3.56 ± 0.09
Hygroscopicity (%)	1.41 ± 0.04	1.02 ± 0.03	0.09 ± 0.02
Degree of caking (%)	1.22 ± 0.05	0.96 ± 0.04	1.72 ± 0.03

### 3.4. FT-IR analysis

The FTIR spectra of Testolift and its individual components are presented in Fig. 3 and summarized in Table 2. Black ginger extract displayed phenolic –OH, methoxy, and aromatic stretching peaks typical of polymethoxyflavones (Fig. 3a),^39,42,43^ while fenugreek extract exhibited broad O–H, glycosidic, and β-linkage bands confirming the presence of steroidal saponins (Fig. 3b).^44–48^ Zinc methionine showed characteristic carboxylate and amino group peaks, with additional Zn–N and Zn–S stretching signals confirming stable metal–ligand coordination (Fig. 3c).^49–52^

**Fig. 3:**
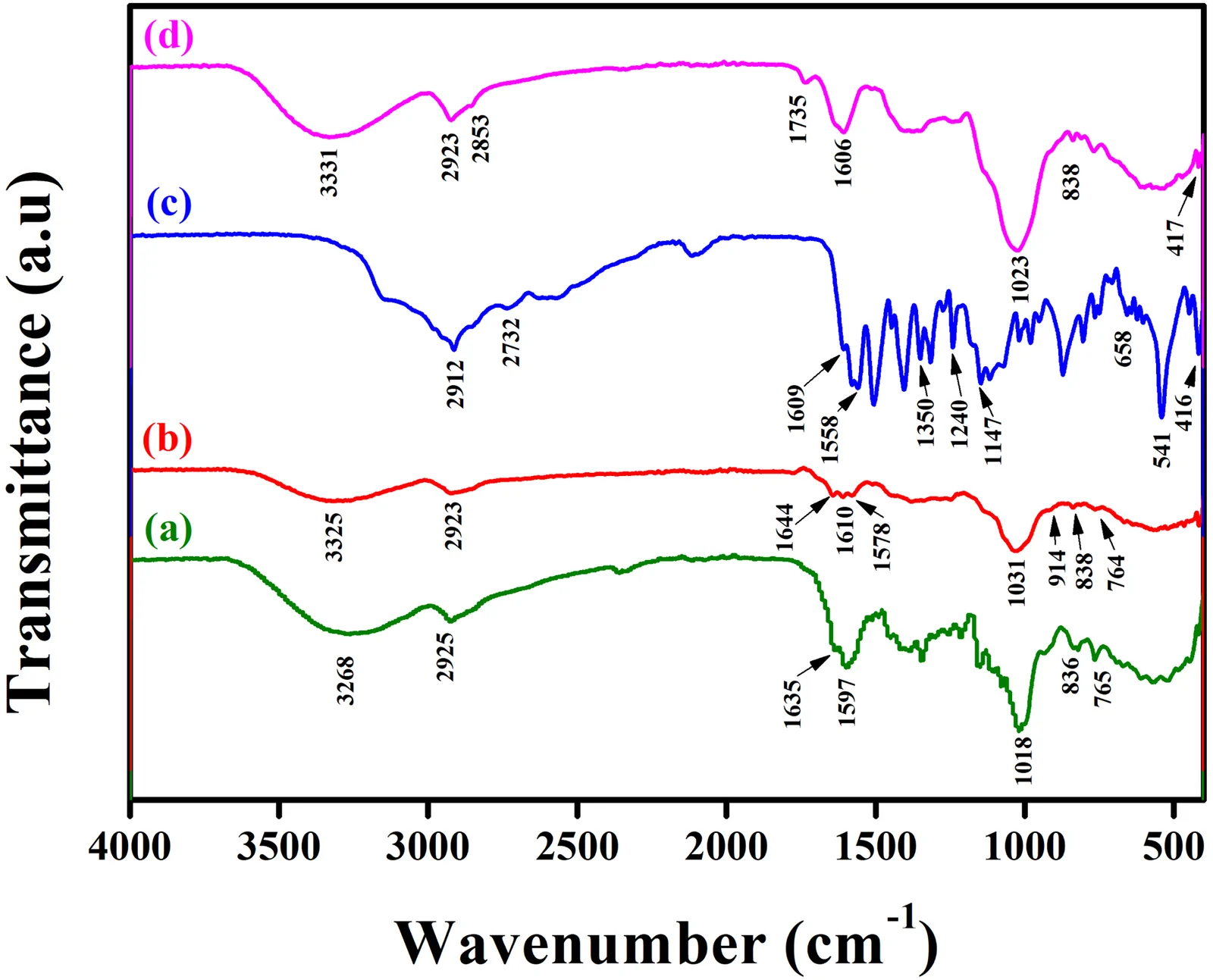
FTIR spectra of (a) black ginger extract, (b) fenugreek seed extract, (c) zinc methionine, and (d) Testolift formulation. Characteristic peaks confirmed the presence of polymethoxyflavones (O–H at 3268 cm^−1^, C=O at 1635 cm^−1^), saponins (O–H at 3325 cm^−1^, β-glycosidic linkages at 914 and 838 cm^−1^), and zinc–methionine coordination (COO^−^ at 1609 cm^−1^, Zn–O/Zn–S at 417 cm^−1^). The final Testolift spectrum showed combined features, including a broad O–H/N–H band at 3331 cm^−1^, ester C=O at 1735 cm^−1^, and Zn–O/Zn–S vibration at 417 cm^−1^, confirming successful integration of all components.

**Table 2:** Major FTIR absorption bands of Testolift components such as black ginger extract, fenugreek seed extract, zinc methionine and Testolift formulation

Sample	Major absorption bands (cm^−1^)	Assignments	Interpretation	Ref.
Black ginger extract (methoxyflavones)	3268 (O–H stretch); 2925 (C–H); 1635, 1597 (C=O); 1513, 1491 (C=C); 1346–1018 (C–O/C–O–C); 836, 821, 765 (C–H out-of-plane)	Phenolic hydroxyls, aliphatic CH, flavonoid carbonyls, aromatic C=C, methoxy substituents	Strong hydrogen bonding, polymethoxylated flavonoids confirmed	39, 42 and 43
Fenugreek extract (saponins)	3325 (O–H); 2923 (C–H); 1644, 1610, 1578 (C=C/C=O); 1511, 1447, 1383 (CH_2_/CH_3_); 1338–1221 (C–O–C); 1138, 1031 (C–O); 914, 838 (β-glycosidic); 812, 783, 764, 667 (skeletal bend); 521–416 (ring modes)	Hydroxyl, glycosidic, aromatic and triterpenoid structures	Confirms polyhydroxylated steroidal/triterpenoid saponins	44–48
Zinc methionine	3246–3117 (N–H/O–H); 2912, 2732 (C–H); 1609–1558 (COO^−^); 1350–1240 (C–N); 1147–1004 (C–O/C–S); 658–602 (Zn–N/Zn–S); 541, 449, 416 (Zn–O/Zn–S)	Amino, carboxylate, and sulfur coordination with zinc	Confirms Zn^2+^ chelation with methionine *via* amino, carboxylate and thioether groups	49–52
Testolift formulation	3331 (O–H/N–H); 2923, 2853 (C–H); 1735 (C=O ester); 1606 (C=C); 1511 (COO^−^); 1407, 1378, 1348 (CH_2_ bend, C–N); 1216, 1023 (C–O/C–N); 838, 810, 769 (C–H bend); 417 (Zn–O/Zn–S)	Hydrogen bonding, aliphatic chains, ester/carboxylate, aromatic, glycosidic, metal–ligand	Confirms successful integration of bioactives, molecular stability, zinc coordination	47, 49, 50 and 53

The final Testolift formulation combined all of these spectral features (Fig. 3d). The strong and broad O–H/N–H peak at 3331 cm^−1^ reflected extensive hydrogen bonding, while aliphatic C–H peaks (2923, 2853 cm^−1^) confirmed lipid and saponin contributions. The ester carbonyl band at 1735 cm^−1^ is particularly important, as it supports the encapsulation of flavonoids within the phospholipid carrier system. Simultaneously, the COO^−^ peak at 1511 cm^−1^ and the Zn–O/Zn–S vibration at 417 cm^−1^ provided direct evidence of zinc–methionine chelation within the formulation.^47,49,50,53^ Overall, the FTIR results confirm the structural integrity, compatibility, and molecular integration of Testolift components, supporting its stability and suitability for nutraceutical applications.

### 3.5. Morphological characterization of Testolift by FE-SEM

Field Emission Scanning Electron Microscopy (FE-SEM) micrographs of Testolift obtained at different magnifications (100×, 1000×, 2500×, and 5000×) revealed distinct morphological features (Fig. 4). At lower magnifications (100× and 1000×), the formulation exhibited a heterogeneous yet uniform distribution of predominantly spherical and few irregular particles. Fine particulates were observed to be embedded or adhered to the larger microspheres, suggesting efficient matrix integration and uniform dispersion of actives achieved during high-pressure homogenization and spray-drying. At higher magnifications (2500× and 5000×), well-defined microspheres with smooth to slightly rough surfaces were evident, ranging from sub-micron to a few microns in diameter. The absence of crystalline aggregates indicates that InSitu360 processing effectively prevented phase separation, supporting complete complexation of fenugreek saponins, black ginger polymethoxyflavones, and zinc methionine within the encapsulating matrix. The morphological uniformity and absence of large irregular clusters are characteristic of stable nano-structured nutraceutical powders optimized for improved flow, dispersibility, and dissolution.^54^ Similar spherical and compact particle morphologies have been reported in spray-dried and nano-encapsulated herbal formulations, where increased surface smoothness and reduced particle aggregation enhance gastrointestinal absorption and oxidative stability.^55,56^ The morphological features observed in Testolift suggests improved dissolution dynamics and better bioaccessibility of its bioactive components, aligning with the physicochemical data and the demonstrated efficacy in rats on serum testosterone levels.

**Fig. 4:**
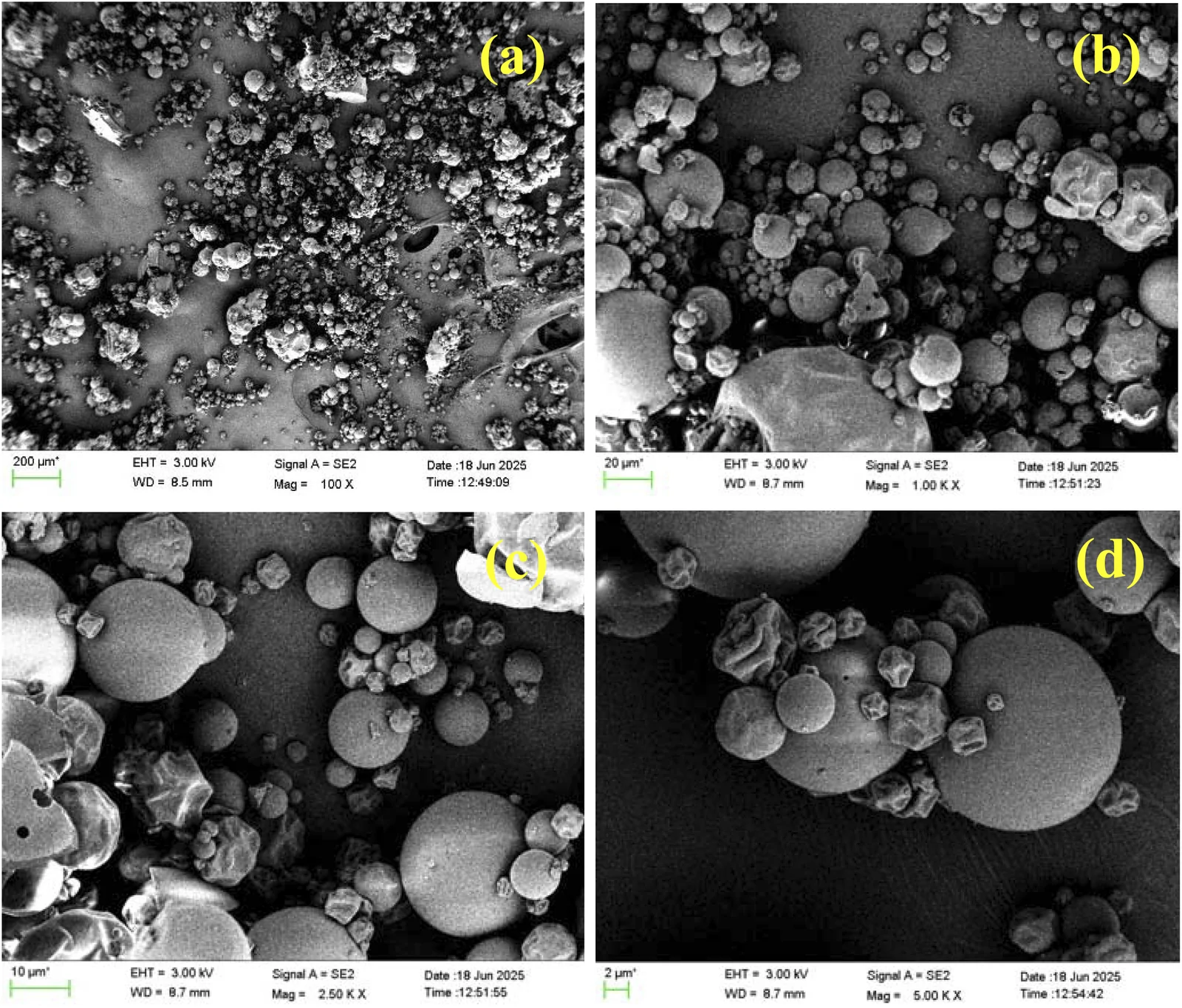
Field emission scanning electron microscopy (FE-SEM) images of the Testolift formulation captured at different magnifications: (a) 100×, (b) 1000×, (c) 2500×, and (d) 5000×. The micrographs reveal uniform spherical and irregular microspheres with smooth surfaces and fine submicron particles adhering to larger structures, indicating efficient dispersion and matrix integration of the bioactives. The absence of crystalline aggregates confirms successful encapsulation and homogeneity achieved through the InSitu360 process.

### 3.6. Elemental composition and mapping by EDS analysis

Energy Dispersive X-ray Spectroscopy (EDS) analysis was conducted to determine the elemental composition and surface distribution of Testolift particles. The EDS spectra and elemental mapping confirmed the presence of the major and trace elements such as carbon, oxygen, nitrogen, sulfur, potassium and zinc (Fig. 5). Carbon and oxygen were the dominant elements, reflecting the organic and plant-based matrix of the formulation. The presence of zinc is attributed to the inclusion of zinc methionine in the formulation, and its detectable level on the surface indicates successful incorporation and stability of the mineral phase in the matrix. Trace elements such as nitrogen and sulfur may originate from amino acid residues (methionine) and bioactive flavonoid components in fenugreek and black ginger extracts. Elemental mapping also indicated a homogeneous distribution of Zn and K across the particle surface, which is critical for consistent release and bioavailability upon ingestion. There was no evidence of elemental clustering or phase segregation. The EDS analysis validates the successful co-localization of both organic and inorganic constituents in the Testolift formulation. The high carbon and oxygen percentages are consistent with polysaccharide, saponin, and flavonoid content in fenugreek and black ginger. The detection of zinc supports its molecular embedding rather than being loosely adsorbed or surface-bound, which is beneficial for improved bioavailability. Importantly, zinc plays a cofactor role in over 300 enzymatic reactions, including steroidogenic enzymes like 17β-HSD, which are critical in testosterone biosynthesis. The uniform zinc distribution confirmed by mapping supports the hypothesis that Testolift may facilitate more effective endocrine support compared to non-chelated zinc salts. Studies have shown that zinc methionine, due to its chelated organic form, exhibits superior intestinal absorption and bio-retention compared to inorganic zinc salts.^51,57,58^

**Fig. 5:**
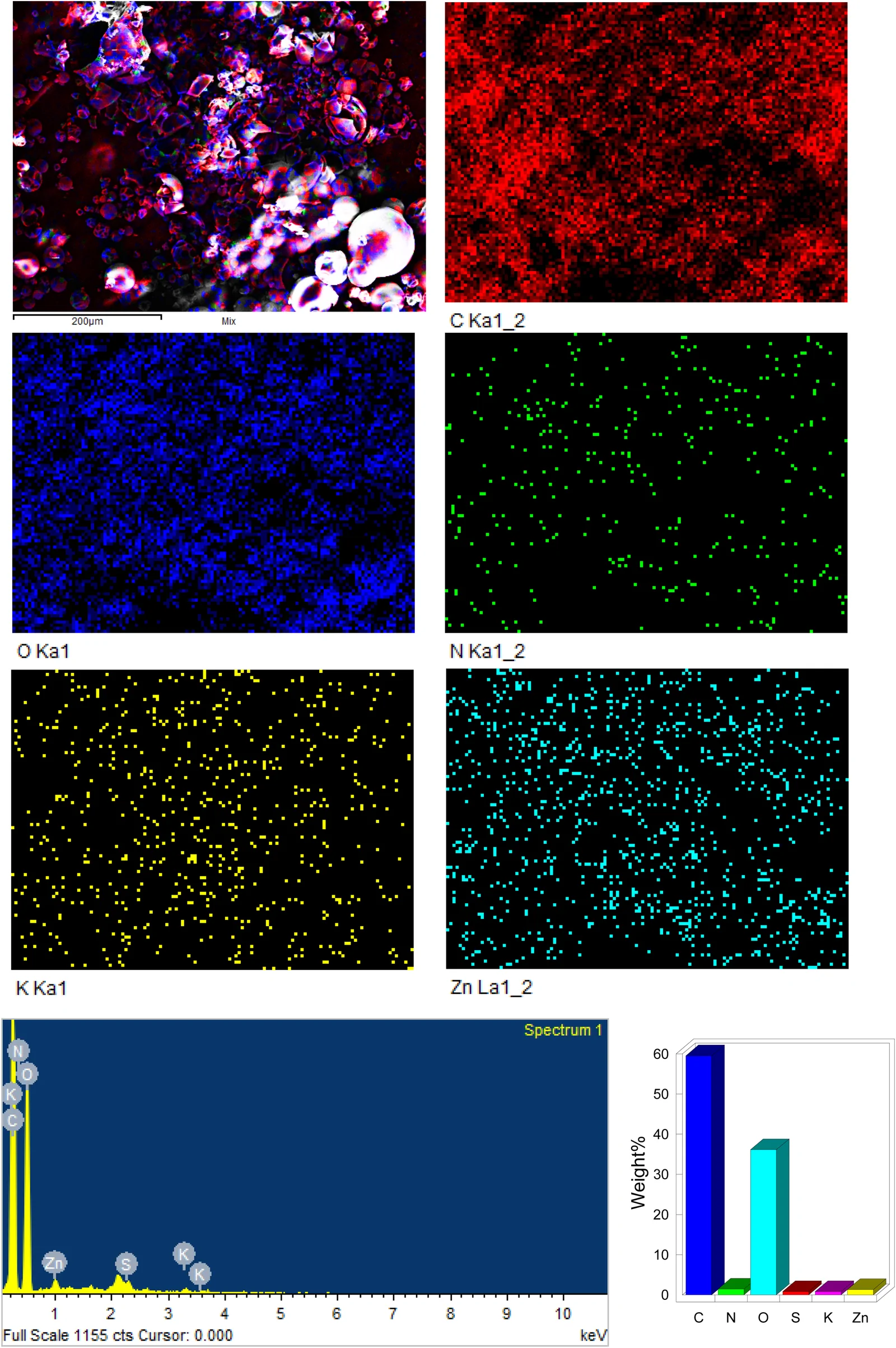
Energy-dispersive X-ray spectroscopy (EDS) spectrum and elemental mapping images of the Testolift formulation. The EDS spectrum confirms the presence of major elements including carbon (C), oxygen (O), nitrogen (N), sulfur (S), potassium (K), and zinc (Zn). Elemental mapping demonstrates a uniform spatial distribution of Zn along with other constituent elements across the formulation matrix, indicating effective incorporation and homogeneous dispersion of zinc methionine within the organic carrier system.

### 3.7. Transmission electron microscopy (TEM) analysis of Testolift

Transmission Electron Microscopy (TEM) was employed to investigate the ultrastructural morphology and particle size distribution of the Testolift formulation. TEM images were captured at magnifications ranging from 50 nm to 1 µm, providing detailed insights into the nanostructure and dispersion of the encapsulated bioactives (Fig. 6). At low magnification (1 µm and 500 nm), the Testolift formulation exhibited a highly dispersed matrix with numerous spherical and ellipsoidal particles embedded within a continuous phase. These particles appeared well-separated, indicating effective prevention of aggregation and uniform dispersion throughout the matrix. At higher magnifications (200 nm and 50 nm), individual nanoparticles were clearly visualized. The particles were predominantly spherical, with diameters ranging from approximately 40 nm to 200 nm. The presence of both isolated and clustered nanoparticles suggests successful encapsulation and stabilization of the active ingredients within the nanomatrix. The dark contrast observed in the core of some particles may indicate dense encapsulation of hydrophobic bioactives, while the surrounding lighter regions suggest a stabilizing shell or matrix material. The absence of large crystalline domains or irregular aggregates further confirms the efficiency of the InSitu360 technology in producing a homogeneous nanostructured formulation. The uniform size distribution and morphology are indicative of synergistic complexation and potential for enhanced bioavailability.

**Fig. 6:**
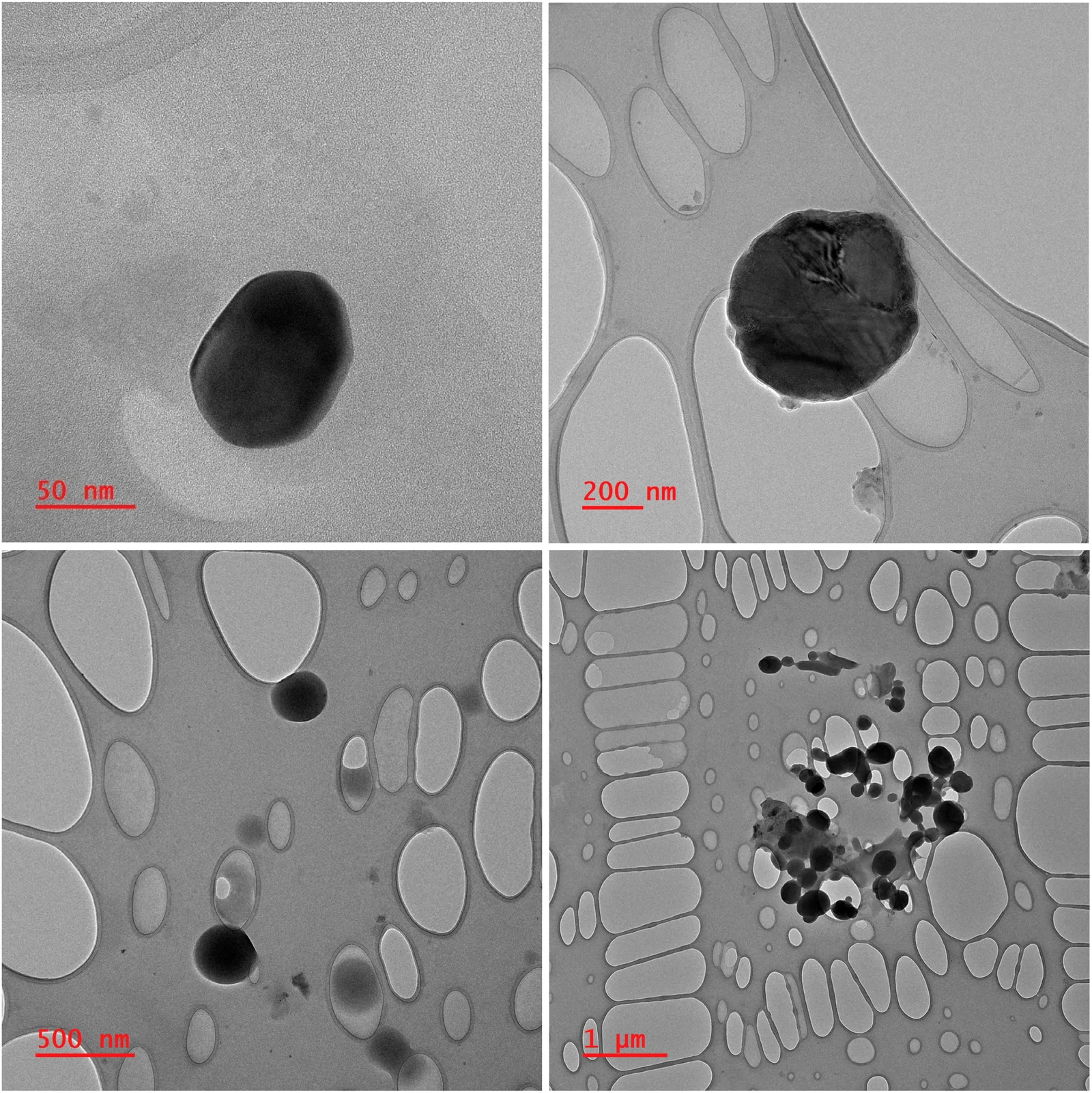
Transmission electron microscopy (TEM) images of the Testolift formulation recorded at varying magnifications, illustrating the morphology and internal structural features of the formulation. Electron-dense regions corresponding to bioactive-loaded domains are observed to be uniformly embedded within a continuous organic matrix, indicating effective encapsulation and dispersion of the bioactive components within the carrier system.

The TEM analysis of Testolift reveals a nanostructured formulation characterized by uniformly dispersed, spherical nanoparticles in the sub-200 nm range. Such morphology is highly desirable for oral nutraceuticals, as nanoscale particles exhibit increased surface area, improved dissolution rates, and enhanced intestinal absorption.^59^ The observed spherical structures and the absence of aggregation suggest that the InSitu360 technology effectively incorporate the bioactive compounds such as fenugreek saponins, black ginger polymethoxyflavones, and zinc methionine within a stable nanomatrix. This complexed incorporation not only protects sensitive actives from degradation but also facilitates controlled release, as supported by previous studies on nanoencapsulated phytochemicals.^60^ Numerous reports have demonstrated that reducing particle size to the nanometer scale substantially increases the oral bioavailability of poorly soluble compounds by improving their dissolution rate and facilitating transcellular and paracellular transport across the intestinal epithelium.^61^ The homogeneous distribution and nanoscale size observed in Testolift are thus expected to translate into superior absorption and systemic delivery of its active components. The uniform nanostructure and lack of crystalline aggregates are particularly important for the stability and reproducibility of nutraceutical formulations. Crystalline domains can impede dissolution and reduce the effectiveness of bioactives, whereas amorphous or nanodispersed systems, as seen here, are associated with improved functional performance.^62^

### 3.8. Particle size distribution and zeta potential analysis

Dynamic light scattering analysis confirmed that the Testolift formulation possessed a particle size distribution in the nanoscale range. The particles were uniformly distributed between 120 nm and 260 nm, with a mean particle size of 193 nm (Fig. 7a). Particle size is a critical characteristic of nutraceutical nanocarriers, as it directly influences their physical stability, chemical stability, solubility, turbidity, release kinetics, and biological performance.^63,64^ The sub-200 nm size observed in Testolift is consistent with favorable biofunctional properties reported for nanosized nutraceuticals and supports the improved dispersibility of the formulation.^65^

**Fig. 7:**
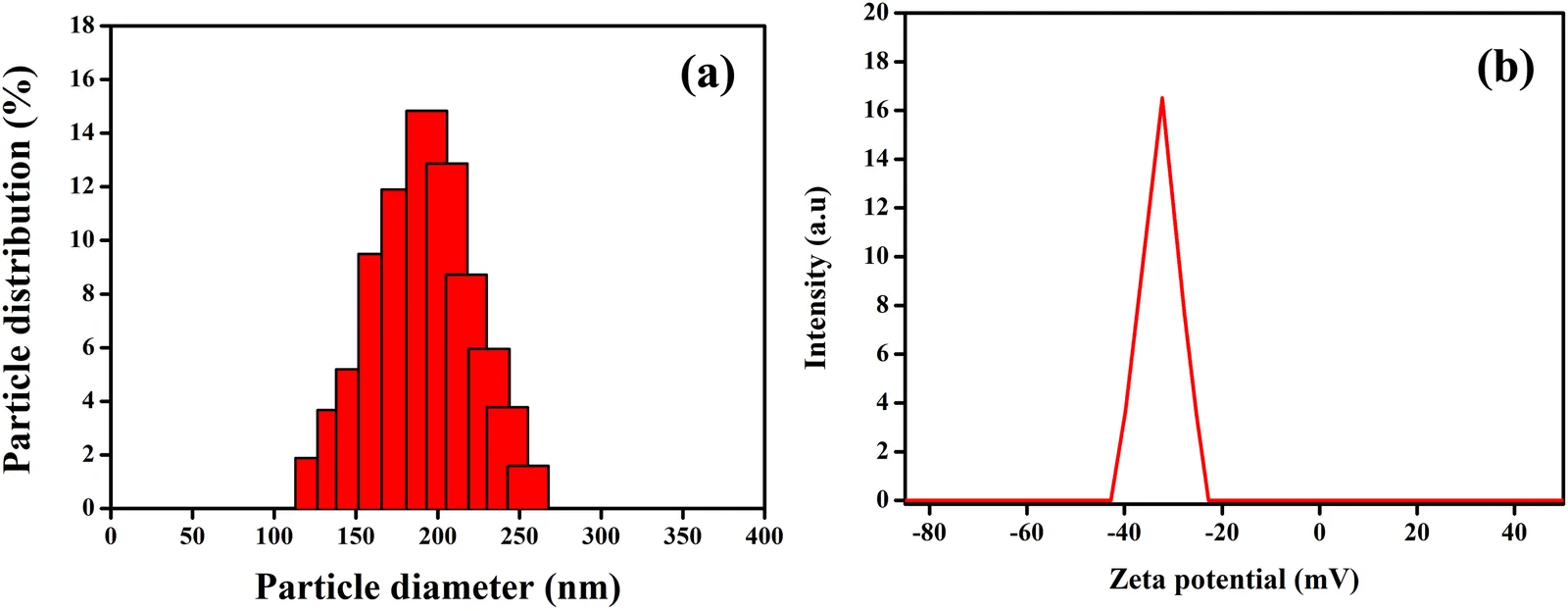
Particle size distribution (a) and zeta potential (b) of Testolift measured by dynamic light scattering. The formulation showed a mean particle size of 193 nm and a zeta potential of −32.24 mV, confirming the stability of the formulation.

The zeta potential of the Testolift nanoparticles was found to be −32.24 mV (Fig. 7b). Zeta potential reflects the surface charge of nanoparticles and provides an indication of the probability of aggregation. A higher absolute zeta potential value (≥|30| mV) is generally considered predictive of electrostatic stabilization and long-term colloidal stability.^66^ The strongly negative charge of Testolift suggests a homogeneous distribution of vesicles and reduced risk of particle aggregation, which can be attributed to the InSitu360 technology employed in the formulation.

### 3.9. DSC analysis

Differential Scanning Calorimetry (DSC) was employed to evaluate the thermal characteristics and intermolecular interactions of black ginger extract, fenugreek seed extract, zinc methionine, and the final Testolift formulation (Fig. 8). The thermogram of black ginger extract (Fig. 8a) displayed two endothermic transitions: a minor event near 86 °C, associated with moisture loss and volatilization, and a broader peak around 174 °C, attributed to the melting or decomposition of methoxyflavones such as 5,7-dimethoxyflavone and 3,5,7-trimethoxyflavone. These results are consistent with previously reported thermal transitions for polymethoxyflavones in the 160–190 °C range.^53^ The fenugreek seed extract (Fig. 8b) showed multiple overlapping endothermic and exothermic transitions between 143 °C and 332 °C, reflecting the breakdown of glycosidic linkages and dehydration of saponin complexes. Such thermal heterogeneity is characteristic of saponin-rich plant matrices due to the presence of carbohydrates and triterpenoid structures.^67^ The thermogram of zinc methionine (Fig. 8c) exhibited a distinct endothermic peak near 73 °C, indicating bound water loss, and a major peak at 233 °C, corresponding to decomposition of the zinc–methionine complex. The high-temperature event confirms the stability and suitability of chelate for thermal processing.^68^

**Fig. 8:**
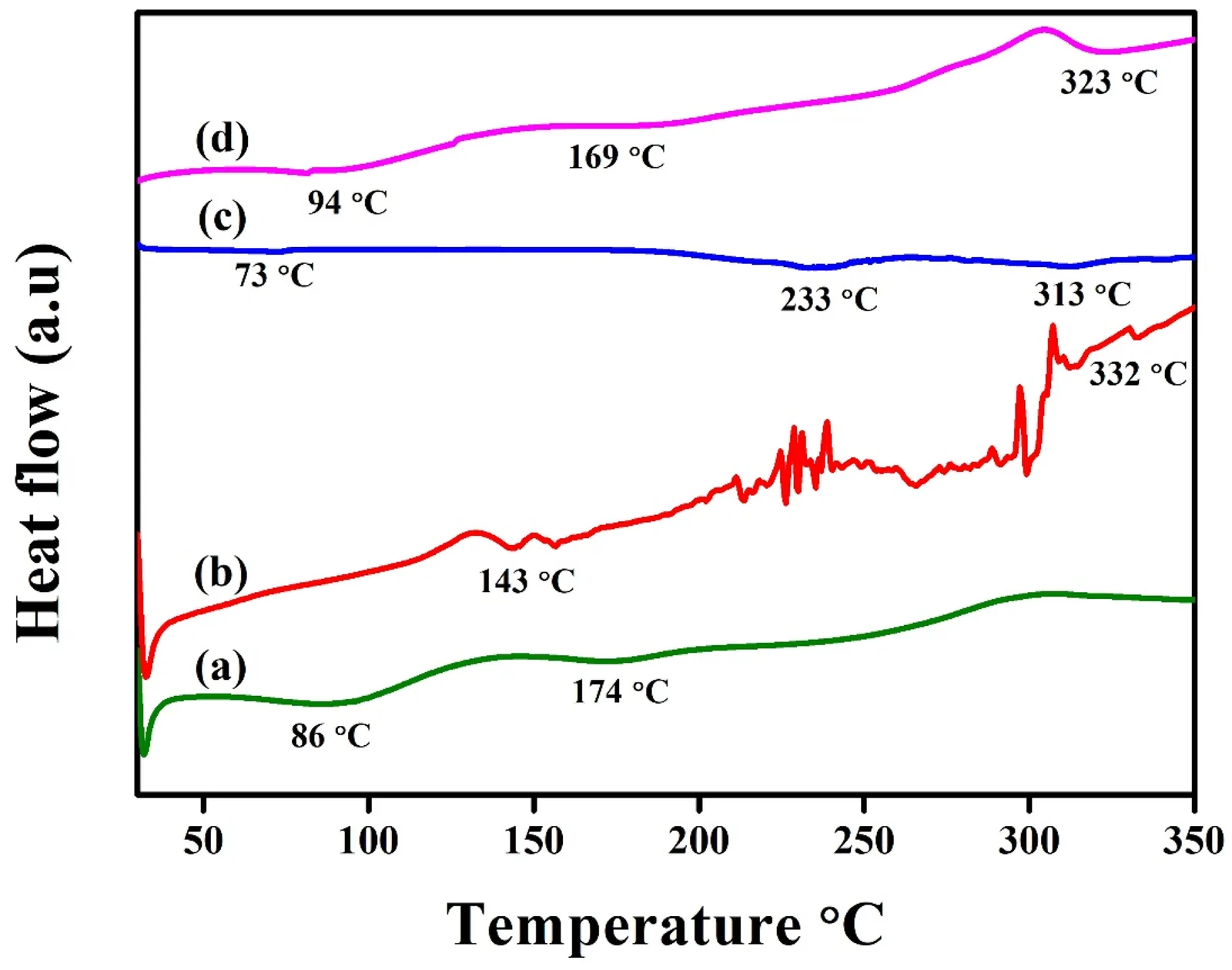
Differential scanning calorimetry (DSC) thermograms of (a) black ginger extract, (b) fenugreek seed extract, (c) zinc methionine, and (d) the Testolift formulation. The thermograms illustrate characteristic thermal transitions of the individual components and the modified thermal profile of Testolift, indicating molecular interaction and integration of the bioactive constituents within the formulation matrix.

In contrast, the composite nutraceutical formulation (Fig. 8(d)) displayed a markedly altered thermal behavior, suggesting significant physicochemical interaction between the constituent bioactives. A broad endothermic peak ranging from 72 °C to 126 °C, with a maximum at 94 °C, was observed, corresponding to bound water release, initial phase transitions, and matrix softening, likely mediated by the interaction of saponins, flavonoids, gum arabic, and phospholipids. The previously distinct peak of methoxyflavones at 174 °C was shifted to 169 °C, while a new thermal transition emerged at 323 °C, indicative of modified thermal stability and enhanced matrix integration. This delayed decomposition temperature suggests improved incorporation, reduced crystallinity, or formation of molecular complexes that improve thermal resistance. The disappearance or broadening of individual peaks from the raw extracts, and the emergence of unified transitions in the composite formulation, indicate molecular-level interactions and co-stabilization effects. Such transitions reflect successful formulation design, with the potential to improve the stability, shelf-life, and processing tolerance of the final nutraceutical product.

### 3.10. Storage stability study of Testolift

The chemical and physicochemical stability of Testolift was examined over an 180 days period under refrigerated, ambient, and accelerated conditions. The results demonstrated that the formulation retained excellent stability throughout the study duration, with all major bioactive constituents showing high levels of retention across the temperature ranges evaluated (Table 3). Methoxyflavones exhibited strong stability across storage conditions, remaining above 97.8% at 4 °C and above 95% even at 45 °C by Day 180. Similar trends were observed for total saponins, which retained more than 98% of their initial concentration at 4 °C and remained above 96% under accelerated conditions. Zinc content was particularly stable, maintaining above 98% retention across all conditions and time points. These findings indicate that Testolift possesses a robust chemical profile resistant to thermal and environmental stress, consistent with the expectations for chelated mineral complexes and microencapsulated phytochemicals.

**Table 3:** Chemical and physicochemical stability of Testolift under refrigerated (4 ± 2 °C), long-term (25 ± 2 °C/60% RH ± 5%), and accelerated (45 ± 2 °C/75% RH ± 5%) storage conditions (mean ± SD, *n* = 3)

Parameters	Initial	30 days	60 days	90 days	120 days	150 days	180 days
**Stability at 4 ± 2°C (refrigerated)**							
Methoxy flavones (%)	99.74 ± 0.38	99.67 ± 0.37	99.11 ± 0.46	98.86 ± 0.41	98.37 ± 0.34	97.79 ± 0.28	97.84 ± 0.36
Saponin content (%)	99.68 ± 0.41	99.51 ± 0.47	99.23 ± 0.36	99.17 ± 0.51	98.79 ± 0.56	98.57 ± 0.45	98.38 ± 0.77
Zinc content (%)	99.89 ± 0.18	99.83 ± 0.22	99.64 ± 0.14	99.41 ± 0.19	99.27 ± 0.24	99.07 ± 0.11	98.92 ± 0.16
Color measurement (*L* × *a* × *b* values)	69.05 ± 0.08, 7.60 ± 0.21, 23.32 ± 2.25	69.02 ± 0.06, 7.58 ± 0.19, 23.30 ± 2.21	68.98 ± 0.07, 7.57 ± 0.17, 23.25 ± 2.23	68.95 ± 0.05, 7.55 ± 0.18, 23.21 ± 2.22	68.92 ± 0.06, 7.54 ± 0.16, 23.17 ± 2.19	68.88 ± 0.07, 7.53 ± 0.18, 23.13 ± 2.17	68.86 ± 0.05, 7.52 ± 0.14, 23.10 ± 2.18
Bulk density (g mL^−1^)	0.37 ± 0.03	0.37 ± 0.04	0.37 ± 0.02	0.36 ± 0.03	0.36 ± 0.03	0.36 ± 0.02	0.36 ± 0.02
Tapped density (g mL^−1^)	0.52 ± 0.02	0.52 ± 0.03	0.52 ± 0.02	0.51 ± 0.04	0.51 ± 0.03	0.51 ± 0.03	0.51 ± 0.04
Water solubility index (%)	6.85 ± 0.09	6.84 ± 0.07	6.83 ± 0.11	6.80 ± 0.08	6.79 ± 0.09	6.78 ± 0.07	6.78 ± 0.08
Moisture content (%)	3.56 ± 0.09	3.57 ± 0.12	3.58 ± 0.08	3.59 ± 0.11	3.60 ± 0.10	3.61 ± 0.08	3.62 ± 0.07
Hygroscopicity (%)	0.09 ± 0.02	0.09 ± 0.03	0.10 ± 0.01	0.10 ± 0.01	0.10 ± 0.02	0.10 ± 0.02	0.11 ± 0.03
Degree of caking (%)	1.72 ± 0.03	1.72 ± 0.05	1.73 ± 0.03	1.73 ± 0.04	1.74 ± 0.02	1.74 ± 0.03	1.75 ± 0.04

**Stability at 25 ± 2°C/60% RH ± 5% (long-term storage)**							
Methoxy flavones (%)	99.74 ± 0.38	99.46 ± 0.45	98.91 ± 0.39	97.72 ± 0.51	97.11 ± 0.49	96.36 ± 0.81	95.75 ± 0.76
Saponin content (%)	99.68 ± 0.41	99.11 ± 0.31	98.92 ± 0.46	98.69 ± 0.54	98.07 ± 0.62	97.71 ± 0.49	97.27 ± 0.59
Zinc content (%)	99.89 ± 0.18	99.76 ± 0.15	99.54 ± 0.21	99.38 ± 0.17	99.22 ± 0.24	99.07 ± 0.19	98.84 ± 0.14
Color measurement (*L* × *a* × *b* values)	69.05 ± 0.08, 7.60 ± 0.21, 23.32 ± 2.25	68.95 ± 0.07, 7.59 ± 0.18, 23.26 ± 2.19	68.88 ± 0.04, 7.58 ± 0.16, 23.20 ± 2.20	68.75 ± 0.06, 7.55 ± 0.17, 23.12 ± 2.18	68.60 ± 0.03, 7.52 ± 0.15, 23.05 ± 2.17	68.52 ± 0.05, 7.51 ± 0.14, 22.97 ± 2.15	68.45 ± 0.04, 7.48 ± 0.13, 22.90 ± 2.14
Bulk density (g mL^−1^)	0.37 ± 0.02	0.37 ± 0.02	0.36 ± 0.03	0.36 ± 0.02	0.36 ± 0.03	0.35 ± 0.03	0.35 ± 0.02
Tapped density (g mL^−1^)	0.52 ± 0.04	0.51 ± 0.03	0.51 ± 0.02	0.51 ± 0.03	0.50 ± 0.02	0.50 ± 0.01	0.50 ± 0.03
Water solubility index (%)	6.85 ± 0.10	6.82 ± 0.08	6.79 ± 0.09	6.77 ± 0.07	6.75 ± 0.09	6.73 ± 0.07	6.70 ± 0.08
Moisture content (%)	3.56 ± 0.08	3.60 ± 0.11	3.63 ± 0.07	3.67 ± 0.08	3.70 ± 0.07	3.73 ± 0.08	3.75 ± 0.06
Hygroscopicity (%)	0.09 ± 0.03	0.10 ± 0.04	0.10 ± 0.03	0.11 ± 0.03	0.11 ± 0.02	0.11 ± 0.03	0.12 ± 0.02
Degree of caking (%)	1.72 ± 0.04	1.73 ± 0.03	1.74 ± 0.04	1.76 ± 0.02	1.78 ± 0.03	1.79 ± 0.02	1.81 ± 0.03

**Stability at 45 ± 2°C/75% RH ± 5% (accelerated storage)**							
Methoxy flavones (%)	99.74 ± 0.38	99.37 ± 0.56	98.73 ± 0.78	97.25 ± 0.48	96.52 ± 0.64	95.88 ± 0.72	95.14 ± 0.83
Saponin content (%)	99.68 ± 0.41	98.86 ± 0.44	98.13 ± 0.58	97.91 ± 0.62	97.53 ± 0.51	97.16 ± 0.47	96.73 ± 0.67
Zinc content (%)	99.89 ± 0.18	99.65 ± 0.59	99.41 ± 0.56	99.13 ± 0.44	98.86 ± 0.78	98.47 ± 0.61	98.02 ± 0.78
Color measurement (*L* × *a* × *b* values)	69.05 ± 0.08, 7.60 ± 0.21, 23.32 ± 2.25	68.82 ± 0.08, 7.56 ± 0.17, 23.15 ± 2.18	68.55 ± 0.06, 7.52 ± 0.19, 23.01 ± 2.21	68.28 ± 0.07, 7.48 ± 0.16, 22.78 ± 2.19	68.10 ± 0.06, 7.44 ± 0.18, 22.63 ± 2.18	67.95 ± 0.08, 7.42 ± 0.15, 22.47 ± 2.17	67.82 ± 0.05, 7.40 ± 0.14, 22.40 ± 2.16
Bulk density (g mL^−1^)	0.37 ± 0.03	0.36 ± 0.03	0.36 ± 0.02	0.35 ± 0.04	0.35 ± 0.02	0.35 ± 0.03	0.34 ± 0.04
Tapped density (g mL^−1^)	0.52 ± 0.02	0.51 ± 0.03	0.51 ± 0.03	0.50 ± 0.02	0.50 ± 0.03	0.49 ± 0.02	0.49 ± 0.03
Water solubility index (%)	6.85 ± 0.08	6.80 ± 0.11	6.75 ± 0.09	6.70 ± 0.09	6.68 ± 0.08	6.65 ± 0.07	6.62 ± 0.08
Moisture content (%)	3.56 ± 0.06	3.65 ± 0.08	3.72 ± 0.07	3.80 ± 0.09	3.87 ± 0.07	3.92 ± 0.08	3.98 ± 0.07
Hygroscopicity (%)	0.09 ± 0.04	0.12 ± 0.03	0.13 ± 0.04	0.14 ± 0.04	0.15 ± 0.05	0.16 ± 0.04	0.17 ± 0.04
Degree of caking (%)	1.72 ± 0.05	1.78 ± 0.04	1.82 ± 0.03	1.87 ± 0.03	1.91 ± 0.04	1.95 ± 0.03	2.00 ± 0.05

Physicochemical parameters also remained largely unchanged during the 180 days period. Colorimetric values (L*, a*, b* values) showed minimal drift, suggesting the absence of oxidative browning or pigment degradation. Moisture content increased only slightly and remained within the acceptable range for spray-dried powder formulations. A gradual increase in moisture content was observed particularly under elevated temperature storage (45 ± 2 °C), which is scientifically expected due to enhanced water vapor permeability, moisture migration, and hygroscopic absorption at higher temperatures. The presence of naturally hygroscopic excipients such as gum arabic and lecithin further contributes to moisture uptake under stressed conditions. Importantly, the observed moisture increase remained within acceptable specification limits and did not adversely impact the chemical stability, physical integrity, or bioactive content of the Testolift formulation.

Hygroscopicity showed negligible change, confirming the moisture-protective function of the gum arabic–lecithin encapsulation matrix. Bulk and tapped densities exhibited minor expected variations, attributable to natural particle settling and compaction during storage. Water solubility index (WSI) and degree of caking values remained consistent across time points, indicating that the powder maintained its dispersibility, flowability, and resistance to agglomeration.

The excellent stability observed can be attributed to the InSitu360 complexation process, which creates a protective matrix around the active ingredients. The gum arabic–lecithin encapsulation system reduces molecular mobility and shields thermolabile components such as polymethoxyflavones and saponins from heat, moisture, and oxidative degradation. These observations are in agreement with recent literature demonstrating that optimized spray-drying and nano-complexation techniques enhance the stability and functional performance of botanical bioactives by promoting amorphous dispersion and reducing crystallinity.^19–23^ Overall, Testolift maintained more than 95% of its chemical constituents and preserved its physicochemical integrity across all tested storage conditions over 180 days. These findings confirm that the InSitu360 technology provides an effective stabilization strategy, producing a formulation suitable for long-term commercial storage and distribution.

The physicochemical characterization of Testolift demonstrated that the InSitu360 process successfully facilitated molecular interactions between fenugreek saponins, black ginger polymethoxyflavones, and zinc methionine. FTIR spectra confirmed the presence of characteristic functional groups with slight peak shifts, indicating molecular interaction. DSC thermograms showed reduced crystallinity and altered thermal transitions, consistent with the formation of a stable amorphous composite. SEM and TEM images further revealed the formation of uniform micro-to nanoscale particles with smooth morphology, supporting the successful encapsulation and stabilization of the bioactives within the gum arabic–lecithin matrix.

Although high inlet temperatures are employed during spray drying, the actual thermal exposure of the bioactives is transient, with outlet temperatures maintained at 75–85 °C, corresponding to the dried particle temperature rather than the feed solution. In addition, encapsulation within the gum arabic–lecithin matrix provides thermal and oxidative protection. Together with rapid solvent evaporation, these conditions preserve the structural integrity of fenugreek saponins, black ginger polymethoxyflavones, and zinc methionine, minimizing degradation and ensuring functional stability. These observations are in agreement with earlier reports showing that optimized spray-drying conditions effectively protect phytoconstituents from thermal breakdown while improving solubility and dispersibility.^20–22^

### 3.11. Microbiological safety assessment of Testolift

The microbiological quality of the Testolift formulation was rigorously evaluated using ISO-standard analytical procedures to confirm its safety for oral consumption. The results demonstrated excellent microbiological quality, with all tested microbial parameters falling well within the acceptable regulatory limits for herbal nutraceutical products. The Total Aerobic Plate Count (TAPC) was found to be <10 CFU per g, indicating an extremely low general microbial load. Similarly, yeasts and molds, coliforms, *Escherichia coli*, and *Staphylococcus aureus* were all detected at levels below the limit of quantification (<10 CFU per g). Importantly, *Salmonella* spp. was completely absent in 25 g of the Testolift sample. These findings confirm that the formulation, processing conditions, and raw material handling under the InSitu360 technology platform are highly effective in controlling microbial contamination. This is particularly critical for botanical-based formulations, which are inherently more susceptible to microbial proliferation due to their natural origin. The demonstrated microbial safety further supports the quality, stability, and regulatory compliance of Testolift for nutraceutical applications.

### 3.12. Effects of Testolift on serum testosterone: results and literature comparison

#### 3.12.1. General health and tolerability

Throughout the 42 days study period, all animals remained healthy, with no mortality or significant clinical symptoms observed in either the control or Testolift-treated groups. Routine cage-side and detailed clinical examinations revealed no abnormalities in appearance, behavior, or physiological parameters. Weekly monitoring of body weight and feed consumption showed no statistically significant differences between the groups, indicating that Testolift administration did not adversely affect general health, growth, or metabolic status (Fig. 9).

**Fig. 9:**
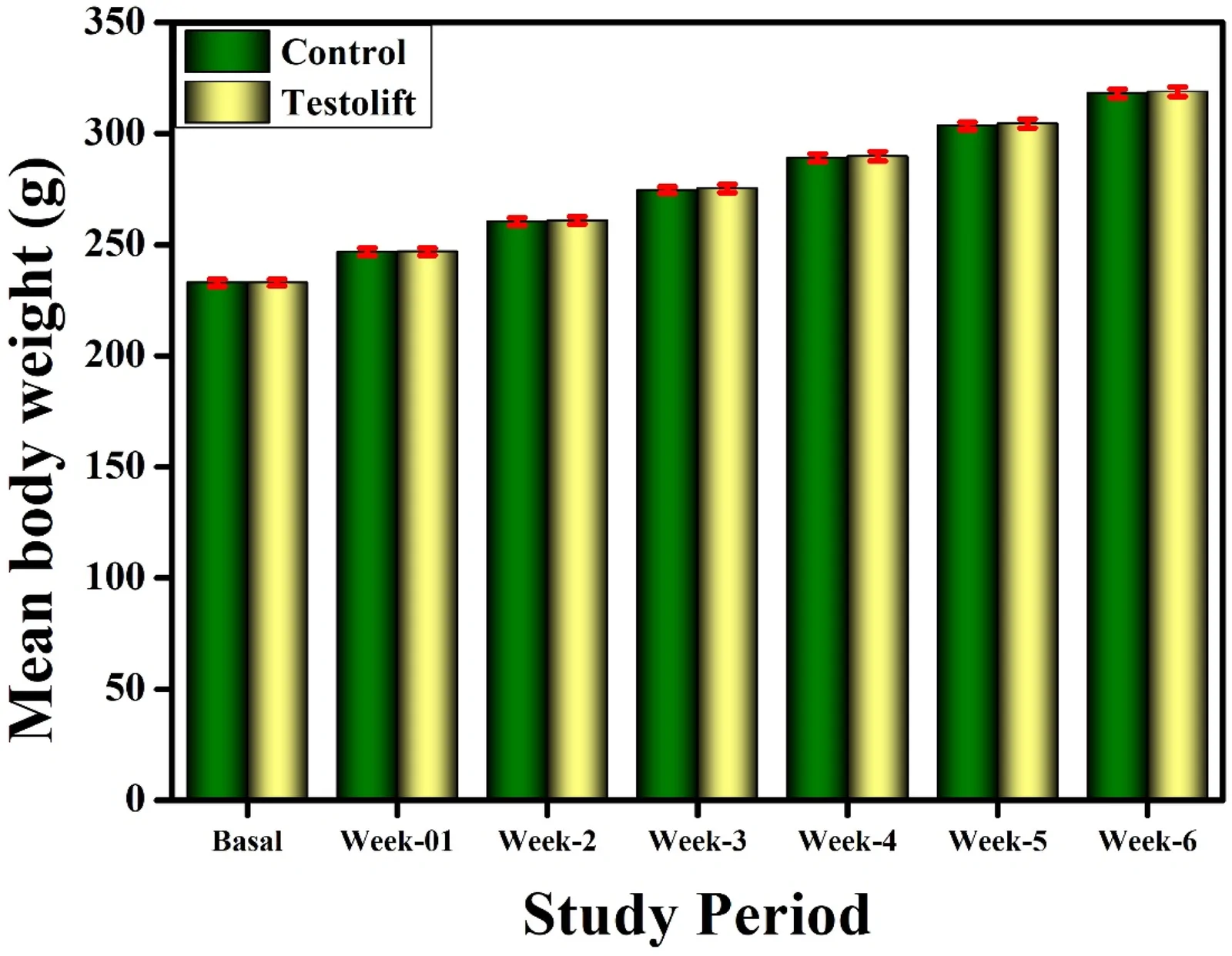
Mean body weight (g) of control and Testolift-treated male Sprague–Dawley (SD) rats during the 42 days oral administration period. Body weight was recorded at baseline and at weekly intervals. Data are expressed as mean ± standard deviation (SD), showing comparable weight gain patterns in both groups throughout the study period.

#### 3.12.2. Effect on serum testosterone levels

Serum testosterone concentrations were measured at baseline (Day 0) and at the end of the study (Day 42) for both groups (Fig. 10). The control group exhibited a modest increase in testosterone levels from 2.808 to 2.924 ng mL^−1^ (+4.13%) over the study period. In contrast, rats receiving Testolift demonstrated a pronounced increase in serum testosterone from 2.793 to 3.428 ng mL^−1^ (+22.74%) from baseline to Day 42. Statistical analysis confirmed that the Testolift group experienced a 18.61% greater increase in testosterone compared to controls, a difference that was statistically significant (*p* < 0.05). No adverse effects or abnormal clinical findings were associated with Testolift treatment, supporting its safety profile in this experimental validation of Testolift in a rodent model.

**Fig. 10:**
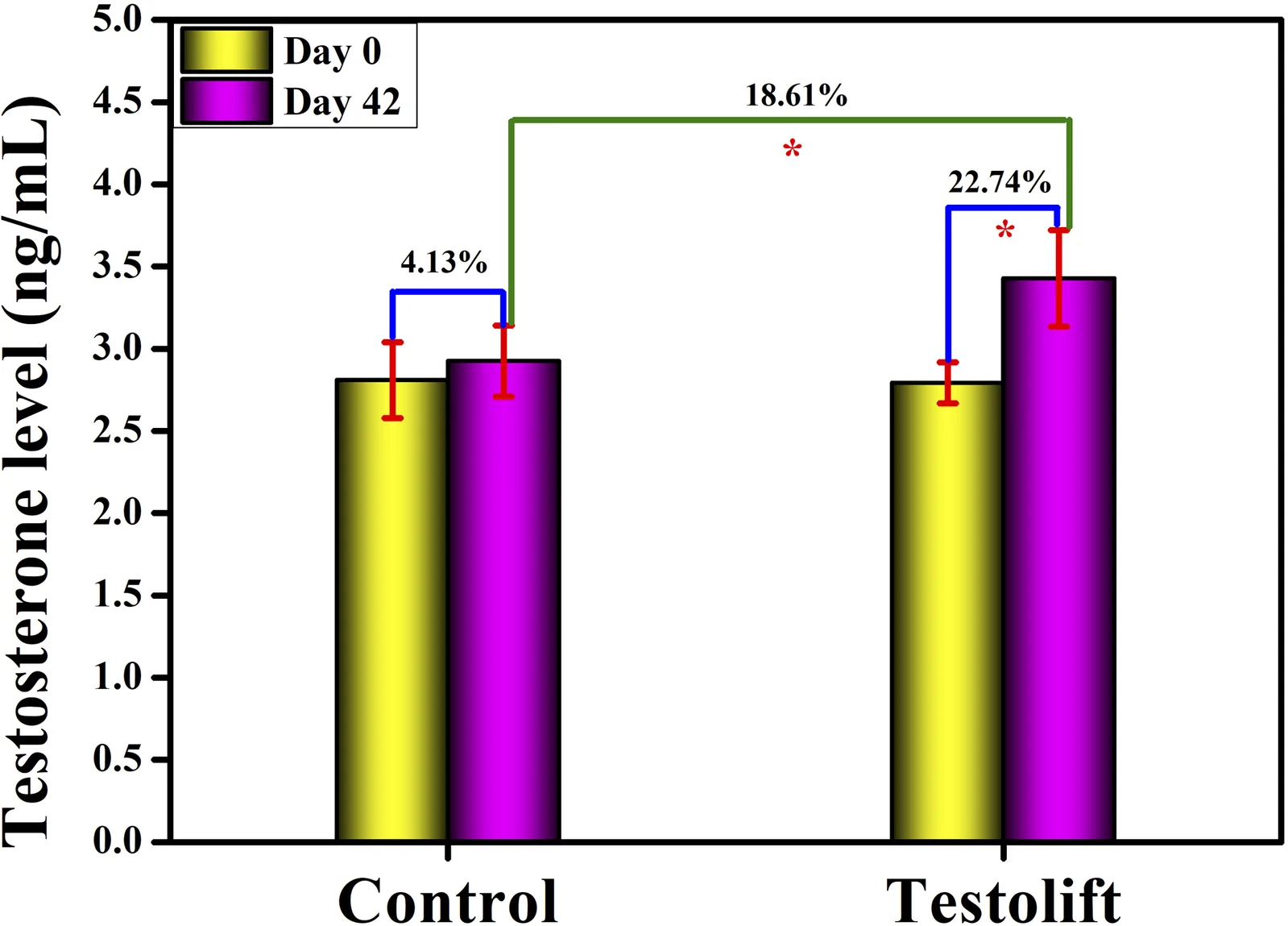
Serum testosterone concentrations (ng mL^−1^) in control and Testolift-treated male Sprague–Dawley rats measured at baseline (Day 0) and after 42 days of oral administration. Data are expressed as mean ± SD. Testolift administration resulted in a significant increase in serum testosterone levels after 42 days compared with baseline and the control group (*p* < 0.05). Percentage changes relative to baseline are indicated above the bars.

#### 3.12.3. Efficacy of Testolift and mechanistic insights

The administration of Testolift, a saponin-enriched nutraceutical formulation containing fenugreek extract, black ginger extract, and zinc methionine resulted in a robust and statistically significant increase in serum testosterone levels in healthy male Sprague Dawley rats. The observed effect size is comparable to or greater than those reported for single-ingredient interventions in the literature, underscoring the potential synergistic benefits of combining these bioactives. Fenugreek extract, rich in steroidal saponins, is known to enhance testosterone synthesis by inhibiting aromatase and 5α-reductase, thereby reducing testosterone conversion to estrogen and dihydrotestosterone. Additionally, fenugreek may upregulate androgen receptors and increase androgen precursors, contributing to its androgenic effects. In addition, fenugreek saponins have been reported to modulate steroidogenic enzymes such as 3β-hydroxysteroid dehydrogenase (3β-HSD) and 17β-hydroxysteroid dehydrogenase (17β-HSD), while also exerting antioxidant protection on Leydig cells against oxidative stress.^69,70^ These mechanisms may explain part of the testosterone-enhancing effect observed in the present study.

Several studies demonstrate that several adaptogenic and phytotherapeutic agents, including *Withania Somnifera* (Ashwagandha), *Eurycoma longifolia* (Tongkat Ali), *Mucuna pruriens*, *Nigella sativa*, and *Trigonella foenum-graecum* (Fenugreek), have been reported to improve testosterone levels, reproductive function, and androgen-related physiological parameters through multiple mechanisms including modulation of the hypothalamic-pituitary-gonadal axis, reduction of oxidative stress, and enhancement of testicular steroidogenesis.^71–73^

Black ginger (*K*. *parviflora*) and related Zingiberaceae family extracts (such as ginger) have demonstrated testosterone-boosting properties in animal models, likely mediated through antioxidant activity, enhancement of nitric oxide and cAMP signaling, and upregulation of testicular steroidogenic enzymes such as CYP11A1.^74,75^ Furthermore, black ginger polymethoxyflavones have shown anti-inflammatory activity through suppression of nitric oxide and pro-inflammatory cytokines, suggesting an additional protective role on testicular and vascular function.^16^ The inclusion of black ginger in Testolift may provide additional benefits for muscle endurance and vascular health, as suggested by improvements in physical performance and sperm parameters in prior studies.

Zinc methionine is a highly bioavailable form of zinc, an essential cofactor for enzymes involved in testosterone biosynthesis and regulation of the hypothalamic-pituitary-gonadal axis. Zinc supplementation is well-established to restore testosterone levels, particularly in zinc-deficient states, and may further potentiate the androgenic effects of Testolift. Taken together, these complementary mechanisms such as aromatase and 5α-reductase inhibition, antioxidant and anti-inflammatory protection, upregulation of steroidogenic enzymes, nitric oxide signaling, and zinc-mediated enzyme cofactor support likely contribute synergistically to the efficacy of Testolift observed in this study.

#### 3.12.4. Comparative analysis with various rat studies

The pharmacological evaluation of black ginger (*K*. *parviflora*), fenugreek (*T. foenum-graecum*), and zinc methionine in various rat models has highlighted their respective roles in supporting male reproductive health and testosterone modulation, which are summarized in Table 4. The Testolift formulation combines these three well-researched components, and its efficacy can be contextualized through comparison with previous studies. Sudwan *et al.*^76^ evaluated oral administration of black ginger extract at doses of 60, 120, and 240 mg kg^−1^ day for 60 days in adult male Wistar rats. While the study found no significant enhancement in sexual behavior, higher doses were associated with increased courtship behavior. Importantly, no adverse effects were noted in body weight, blood parameters, or histopathological findings, suggesting safety even at elevated doses. Chaturapanich *et al.*^77^ further demonstrated that ethanolic black ginger extract (70 mg kg^−1^ day^−1^) significantly reduced sexual latencies and enhanced testicular blood flow in adult rats. While no significant changes were seen in sperm motility or fertility rate, the improved vascularity may support testicular function indirectly. In contrast, Lert-Amornpat *et al.*^78^ showed more pronounced benefits of black ginger in a diabetic model, with significant improvements in serum testosterone and sexual performance. Similarly, Trisomboon *et al.*^79,80^ confirmed that black ginger administration increased serum testosterone and reduced stress biomarkers such as corticosterone in Wistar-Imamichi rats, without adverse reproductive or behavioral effects. Ong *et al.*^81^ explored the protective effects of black ginger against fenitrothion-induced reproductive toxicity. Their study concluded that black ginger restored testosterone, sperm count, motility, and viability in exposed animals, highlighting its adaptogenic potential.

**Table 4:** Comparison of testosterone boosting efficacy of different formulations with Testolift

Intervention Details	Animal types	Age (approx.)	Group size	Study period	Important findings	Ref.
Oral ethanolic extract of black ginger, doses: 60, 120, 240 mg kg^−1^ day^−1^	Adult male Wistar rats	8–12 weeks	8 rats per group	60 days (2 months)	No enhancement of sexual behavior; high dose reduced courtship behavior. No significant toxicity in body weights or blood parameters; subtle liver histological changes at highest dose. Lower doses safe without adverse effects	76
Oral extracts: alcohol, hexane, water of black ginger; 70 mg kg^−1^ day^−1^ dose (main findings with alcohol extract)	Adult male rats	10–12 weeks	8 rats per group	3–5 weeks	Alcohol extract of black ginger significantly reduced sexual latencies and increased testicular blood flow; no effect on fertility rate or sperm motility; no effect on reproductive organ weights; no toxicity observed	77
Oral ethanolic extract of black ginger, doses: 140, 280, 420 mg kg^−1^ day^−1^	Adult male Wistar rats	6–8 weeks	5 rats per group, total 25	6 weeks	Black ginger extract improved sexual behavior and serum testosterone in diabetic rats; highest dose increased sperm density. No effect on blood glucose or body weight	78
Oral gavage, 1000 mg kg^−1^ day^−1^ ethanolic extract of black ginger	Adult male Wistar-Imamichi rats	10–12 weeks	8 rats per group	45 days	No adverse effect on reproductive organ weights, mating behavior or reproductive hormones; decreased corticosterone levels possibly indicating stress reduction. Body weight increased	79
Oral gavage of 1000 mg kg^−1^ day^−1^ ethanolic extract of black ginger	Castrated immature male Wistar-Imamichi rats	3–4 weeks (prepubertal)	7–8 rats per group	5 consecutive days	Serum testosterone significantly increased; androgen-dependent organs showed non-significant trend to increase; no significant androgenic tissue growth. Body weight gain transiently increased	80
Oral black ginger extract (100–200 mg kg^−1^ day^−1^) + fenitrothion (20–40 mg kg^−1^ day^−1^); fenitrothion induces toxicity	Adult male mice	8–10 weeks	6–8 mice per group	28 days (4 weeks)	Black ginger extract protected against fenitrothion-induced reproductive toxicity by restoring testosterone, sperm count, motility, and viability	81
Oral administration of furostanol glycosides fraction (Fenu-FG) at doses of 10 and 35 mg kg^−1^ day^−1^	Immature castrated male Wistar rats; also non-castrated immature rats	55 ± 5 g (weight)	6–8 rats per group	4 weeks	Fenu-FG (35 mg kg^−1^) increased levator ani muscle weight and body weight, showing anabolic activity without androgenic activity (no increase in testosterone). Fenu-FG did not elevate serum testosterone in castrated rats but induced anabolic effects. No toxicity observed	82
Oral administration of fenugreek seed extract; 100–300 mg kg^−1^ day^−1^	Adult rats	Adult	Not specified	Not specified	Fenugreek seed extract improved lipid profiles, reduced fat accumulation, enhanced glucose metabolism, and showed anti-inflammatory activities. Contains steroidal glycosides (*e.g.*, diosgenin). Supports metabolic health with potential indirect benefits on reproductive health through fat reduction and hormone regulation. Showed safety in subchronic administration	83
Rats were fed a zinc-deficient diet to induce zinc deficiency. Some groups received exogenous testosterone supplementation to assess effects on testosterone synthesis and skeletal muscle in zinc-deficient conditions	Male Sprague Dawley rats	Not specified	Not specified	6 weeks	Zinc deficiency significantly lowers serum testosterone levels by impairing testicular steroidogenesis and Leydig cell function, which reduces endogenous testosterone synthesis	84
					Zinc deficiency negatively affects skeletal muscle health through decreased testosterone levels and impaired anabolic signaling, highlighting the essential role of zinc in maintaining male reproductive and muscular function	
Testolift formulation containing fenugreek extract (rich in steroidal saponins), *Kaempferia parviflora* extract (black ginger), and zinc methionine; dose: 30 ± 0.5 mg kg^−1^ body weight once daily for 42 consecutive days	Male Sprague Dawley rats	10 weeks old; body weight range 250–280 g	16 rats total, randomized into 2 groups: control (*n* = 8), Testolift (*n* = 8)	42 days (6 weeks) of daily administration	At day 42, serum testosterone increased by +22.74% from baseline in Testolift group compared to a +4.13% increase in controls; Testolift group showed a statistically significant 18.61% greater increase in serum testosterone relative to control (*p* < 0.05)	Current study

Fenugreek extracts and their steroidal saponins have also been validated. Aswar *et al.*^82^ demonstrated increased lean muscle mass and body weight in immature rats without raising serum testosterone levels, suggesting anabolic benefits through non-androgenic pathways. Kumar *et al.*^83^ further supported the metabolic and anti-inflammatory benefits of fenugreek, which may secondarily improve reproductive performance. Zinc deficiency is known to reduce serum testosterone *via* impaired testicular steroidogenesis. Ai *et al.*^84^ showed that dietary zinc repletion reversed testosterone suppression and supported muscle growth by enhancing androgen receptor sensitivity and Leydig cell function.

Testolift, a multi-component formulation combining standardized extracts of black ginger, fenugreek, and zinc methionine, was tested in male Sprague Dawley rats. Administered at 30 ± 0.5 mg kg^−1^ body weight for 42 days, Testolift significantly increased serum testosterone by 22.74% from baseline and by 18.61% compared to controls (*p* < 0.05). This effect appears to stem from the synergistic activity of its constituents targeting testosterone synthesis, stress reduction, muscle metabolism, and hormonal modulation. Testolift provides an integrative strategy that combines the well-established bioactivities of *K*. *parviflora*, *T*. *foenum-graecum*, and zinc methionine. Comparative studies show that while individual agents exhibit specific benefits ranging from increased vascularization and lean muscle gain to testosterone restoration, Testolift uniquely integrates these mechanisms. Its multifaceted action makes it a scientifically grounded candidate for supporting male reproductive health, testosterone enhancement, and anabolic maintenance, making it suitable for use in functional foods, dietary supplements and various nutraceutical applications. Furthermore, the individual bioactive components of Testolift have independently demonstrated benefits for muscle performance and male health beyond testosterone modulation alone. Fenugreek saponins have been shown to support anabolic activity and lean muscle mass in both animal and human studies.^11,70^ Black ginger polymethoxyflavones have demonstrated improvements in physical fitness performance and muscular endurance through anti-inflammatory and mitochondrial energy-enhancing mechanisms.^16^ Zinc methionine is established as an essential cofactor for anabolic hormone signaling and muscle protein metabolism.^85^ The integration of these three bioactives in Testolift thus provides a multi-mechanistic basis for muscle support claims that extends beyond serum testosterone alone.

### 3.13. Contextual comparison of Testolift with testosterone replacement therapy (TRT): a literature-based perspective

Testosterone replacement therapy (TRT) is the standard clinical intervention for the management of hypogonadism and age-related testosterone deficiency. Clinical studies consistently show that TRT produces rapid and substantial increases in serum testosterone, often restoring levels to the mid-to-upper physiological range.^86^ However, TRT is also associated with several documented risks, including suppression of endogenous testosterone production, testicular atrophy, infertility, erythrocytosis, cardiovascular risk amplification, sleep apnea exacerbation, and prostate-related complications. Long-term TRT has also been linked with hypothalamic-pituitary-gonadal (HPG) axis suppression, necessitating continuous therapy for maintenance of testosterone levels.^86,87^ In contrast, nutraceutical-based testosterone-support formulations such as Testolift are designed to stimulate endogenous testosterone biosynthesis rather than replace the hormone exogenously. The observed increase in serum testosterone in rats treated with Testolift reflects activation of physiological steroidogenic pathways, likely mediated through the combined actions of fenugreek saponins (luteinizing hormone stimulation and 5α-reductase modulation), black ginger polymethoxyflavones (enhancement of mitochondrial activity and nitric oxide signaling), and zinc methionine (cofactor support for key steroidogenic enzymes).^16,85,88^

Compared with TRT, which delivers supraphysiological testosterone concentrations in many cases, nutraceutical interventions generally produce moderate but physiologically regulated elevations in testosterone. While the absolute magnitude of testosterone increase with nutraceuticals is typically lower than that achieved with TRT, the resulting hormonal modulation is more stable, self-regulated, and associated with a superior safety profile. Importantly, nutraceutical approaches do not suppress spermatogenesis or endogenous gonadotropin secretion, preserving reproductive function an established limitation of TRT.^85,88^ From a safety standpoint, TRT requires close clinical monitoring due to risks associated with long-term androgen exposure.^86,87^ In contrast, Testolift demonstrated no adverse effects, no abnormal clinical signs, and stable body weight and feed intake during the 42 days *in vivo* evaluation. Although long-term toxicological evaluation remains necessary, these findings support the favorable biological tolerance of the formulation under physiological conditions. Thus, while TRT remains the most potent therapeutic strategy for severe hypogonadism, nutraceutical formulations such as Testolift represent a safer, non-pharmacological alternative for individuals with age-related testosterone decline, functional hypogonadism, metabolic-associated androgen deficiency, and those seeking long-term hormonal support without endocrine suppression. Direct comparative studies between Testolift and TRT are warranted to quantitatively establish equivalency or complementary benefits under controlled experimental and clinical settings.

### 3.14. Limitations and future directions

While the present study demonstrated that Testolift significantly increased serum testosterone levels *in vivo*, additional physiological outcomes such as muscle gain, lean muscle mass relative to body weight, and metabolic activity were not assessed. These endpoints are important for fully characterizing the functional benefits of testosterone enhancement. Future preclinical and clinical investigations are planned to include comprehensive body composition and metabolic assessments, which will provide a clearer understanding of the role of the formulation in supporting muscle performance and overall male health. Future investigations including single-ingredient treatment arms and formal synergy assessments will be necessary to determine the relative contribution of each component and to confirm whether the observed benefits result from additive or synergistic interactions and investigation of broader endocrine and reproductive effects of Testolift through a more comprehensive biomarker panel and tissue-level analyses will be studied in future. Furthermore, inclusion of a testosterone replacement therapy (TRT) reference group in subsequent studies will enable direct comparative evaluation of the efficacy of Testolift against standard pharmacological interventions. Such comparative studies, under the NIMP platform, will help determine whether the nutraceutical offers equivalent or superior benefits with a safer physiological profile.

## 4. Conclusion

In conclusion, the present study successfully formulated and characterized Testolift, a saponin–methoxyflavones enriched testosterone-boosting nutraceutical integrating fenugreek extract, black ginger extract, and zinc methionine using InSitu360 molecular complexation technology under the Natural Ingredients for Mental and Performance (NIMP) Platform. Comprehensive analytical characterization confirmed the presence and stable incorporation of key bioactive constituents, including polymethoxyflavones, elemental zinc, and saponins, within the formulation matrix. Advanced morphological analyses (FE-SEM with EDS and TEM) revealed a nanostructured and well-dispersed system, indicating effective integration of bioactives within the carrier network. Thermal and FTIR analyses demonstrated favorable stability and intermolecular compatibility within the matrix. The 42 days *in vivo* efficacy study in male rats showed that Testolift significantly enhanced serum testosterone levels compared to controls, with no adverse effects on health parameters. The observed increase in testosterone aligns with the mechanisms of action of fenugreek saponins, black ginger methoxyflavones, and zinc methionine, each known to support endocrine function, antioxidant status, and anabolic pathways. Additionally, Testolift exhibited favorable physicochemical traits such as reduced hygroscopicity, improved solubility, and consistent color stability attributes essential for shelf-life and product integrity. These findings collectively support the formulation stability and biological efficacy of Testolift as a natural testosterone-support formulation. Further clinical investigations are warranted to confirm its translational relevance and long-term benefits in human populations.

## Author contributions

Augustine Amalraj: conceptualization, data curation, investigation, methodology, validation, visualization, writing – original draft, writing – review & editing. Kaniyath Ramachandran Reshna and Karthik Varma: data curation, resources, visualization. Ann Mariya Jogy: data curation, validation. Preetha Balakrishnan: conceptualization, data curation, investigation, methodology, validation, visualization, writing – review & editing. Sreeraj Gopi: conceptualization, data curation, funding acquisition, investigation, methodology, supervision, validation, visualization, writing – review & editing.

## Conflicts of interest

The authors declare that there is no conflict of interest.

## Supplementary Material

RA-016-D6RA02748B-s001

## Data Availability

All data supporting this study are available in the manuscript. Additional raw data may be requested from the corresponding author. Supplementary information (SI) is available. See DOI: https://doi.org/10.1039/d6ra02748b.
