# Supplementary material for: Formulation, characterization, and efficacy evaluation of testolift: a novel testosterone-enhancing nutraceutical featuring synergistic black ginger methoxyflavones, fenugreek saponins, and zinc methionine via InSitu360 technology
Source: RSC Adv. 2026 Jul 2;16(47):61700–25. doi: 10.1039/d6ra02748b (PMC13326673; doi:10.1039/d6ra02748b)
Supplement: Supplementary file 1 [file RA-016-D6RA02748B-s001.pdf]

Supplementary Information (SI) for RSC Advances.
This journal is © The Royal Society of Chemistry 2026

Supporting Information

Formulation, Characterization, and Efficacy Evaluation of Testolift: A Novel Testosterone-Enhancing Nutraceutical Featuring Synergistic Black Ginger Methoxyflavones, Fenugreek Saponins, and Zinc Methionine via InSitu360 Technology

Augustine Amalraj,[a,b,c,d] Kaniyath Ramachandran Reshna,[a,b,c,d] Karthik Varma,[a,b,c,d] Ann Mariya Jogy,[c,d] Preetha Balakrishnan,[a,b,c,d] Sreerag Gopi[a,b,c,d]*

[a]NIMP Innovation Hub, Padmalife Nutrition Private Limited, Koratty, Thrissur - 680 309, Kerala, India.

[b]Innovation Centre, Padmalife Nutrition Private Limited, Mahatma Gandhi University Innovation Foundation, Building No. 10/572, Priyadarshini Hills, Athirampuzha, Kottayam, Kerala 686560, India

[c]Global Innovation Centre, Molecules Biolabs Private Limited, Koratty, Thrissur - 680 309, Kerala, India.

[d]Atomin Innovations Private Limited, Meloor, Thrissur – 680311, Kerala, India

Corresponding Author: *sreeraggopi@gmail.com

S1. System Suitability and Peak Purity

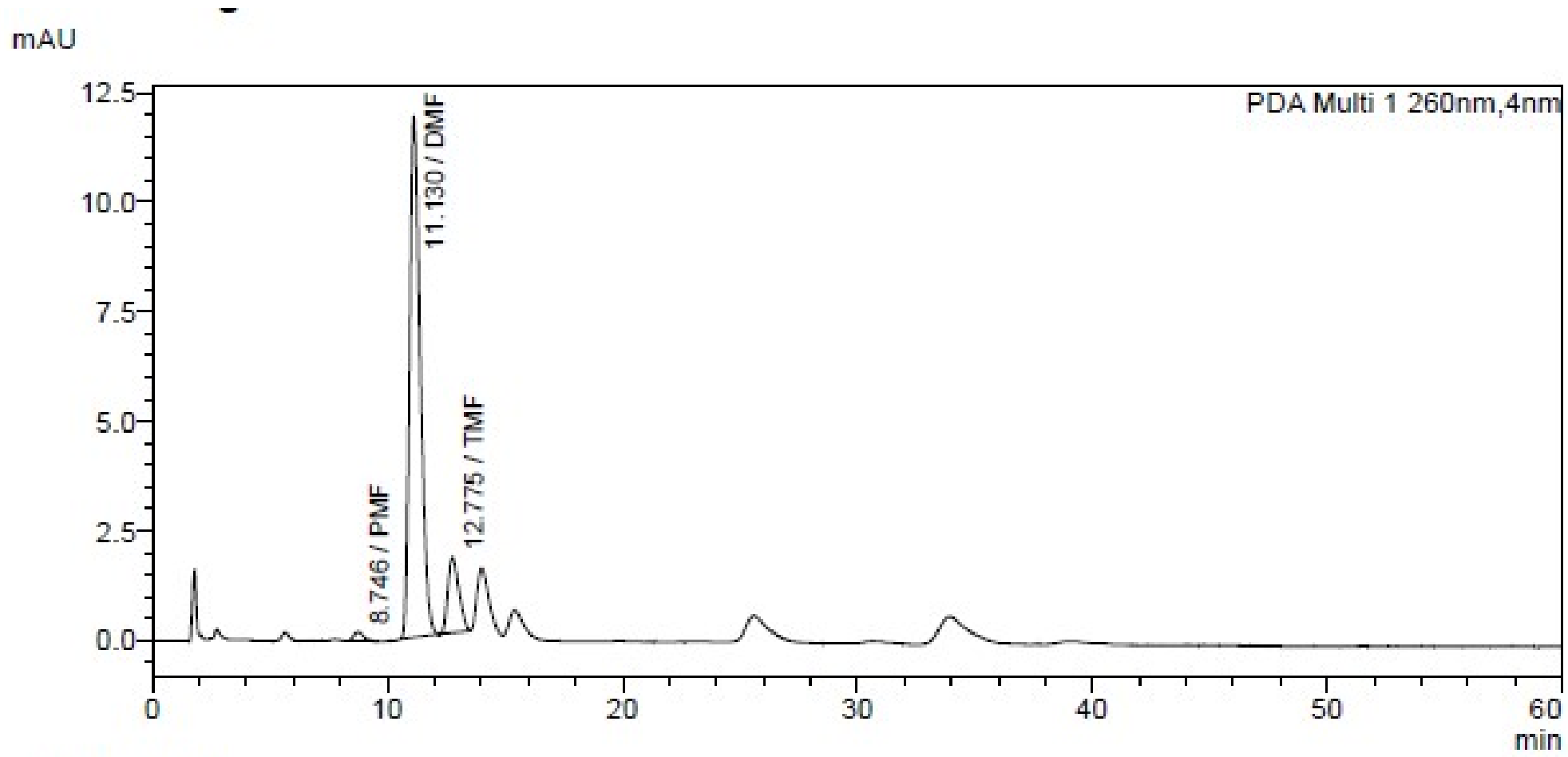

<Peak Table>

PDA Ch1 260nm

Peak#	Ret. Time	Area	Height	Conc.	Unit	Mark	Name
1	8.746	5894	206	0.000	mg/L	M	PMF
2	11.130	376613	11893	0.000	mg/L	M	DMF
3	12.775	56417	1721	0.000	mg/L	M	TMF
Total		438924	13821				

Number of Theoretical Plate(USP)	Tailing Factor	Resolution(USP)
2179	1.493	--
2808	1.367	3.000
3231	1.280	1.892

Peak Purity Index
-0.144090
-0.017312
0.054997

Figure S1. Representative system suitability UPLC chromatogram of the polymethoxyflavone (PMF) mixed standard acquired using a photodiode array (PDA) detector under the chromatographic conditions described in Section 2.3.1, showing well-resolved peaks corresponding to PMF, DMF, and TMF.

Table S1. System suitability parameters for polymethoxyflavone (PMF) standards evaluated in accordance with ICH Q2(R1).

Analyte	Retention Time (min)	Theoretical Plates (N)	USP Tailing Factor (T)	Resolution (Rs)*	Peak Purity
PMF	8.746	≥ 2000	≤ 2.0	—	Acceptable
DMF	11.130	≥ 2000	≤ 2.0	≥ 1.5	Acceptable
TMF	12.775	≥ 2000	≤ 2.0	≥ 1.5	Acceptable

*Resolution (Rs) is calculated only between adjacent chromatographic peaks. Rs is not applicable for the first eluting peak.

Peak Purity Assessment.

Peak purity was assessed using the PDA detector integrated within the UPLC system, as part of routine chromatographic analysis. The PMF, DMF, and TMF peaks exhibited consistent PDA spectral profiles across the peak, with no evidence of co-eluting components. The peak purity indicators generated by the LabSolutions software were within predefined acceptable limits, confirming chromatographic specificity and suitability of the method for quantitative analysis.

All evaluated system suitability parameters met the predefined acceptance criteria, confirming suitability of the chromatographic system for quantitative analysis.

S2. Calibration for linearity

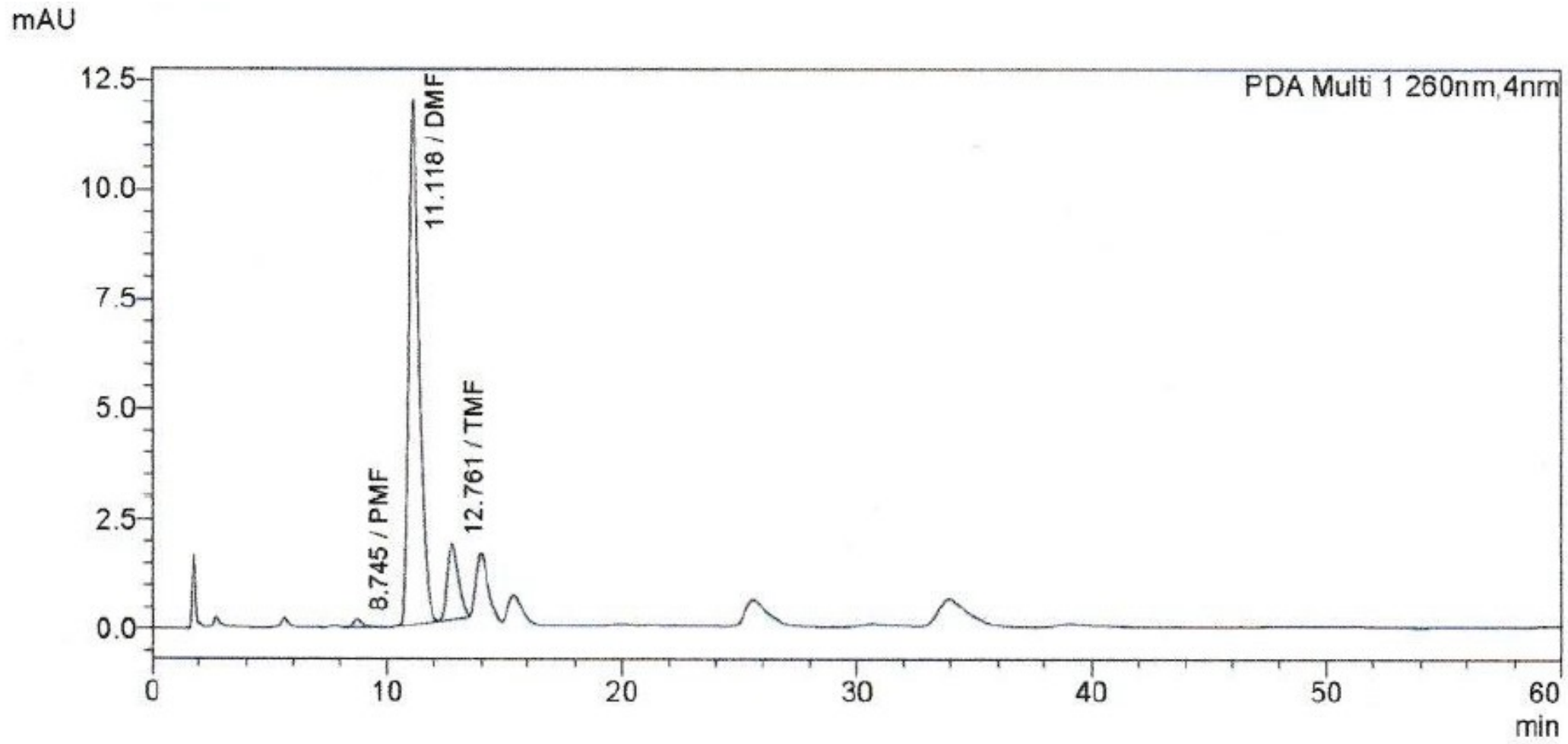

<Peak Table>

PDA Ch1 260nm

Peak#	Ret. Time	Area	Height	Conc.	Unit	Mark	Name
1	8.745	5529	201	0.000	mg/L	M	PMF
2	11.118	380358	11953	0.000	mg/L	M	DMF
3	12.761	57287	1733	0.000	mg/L	M	TMF
Total		443174	13887				

Figure S2. UPLC of major polymethoxyflavones (PMFs) such as 5,7-dimethoxyflavone (DMF), 5,7,4'-trimethoxyflavone (TMF), and 3,5,7,3',4'-pentamethoxyflavone (PMF) at 10 mg/L

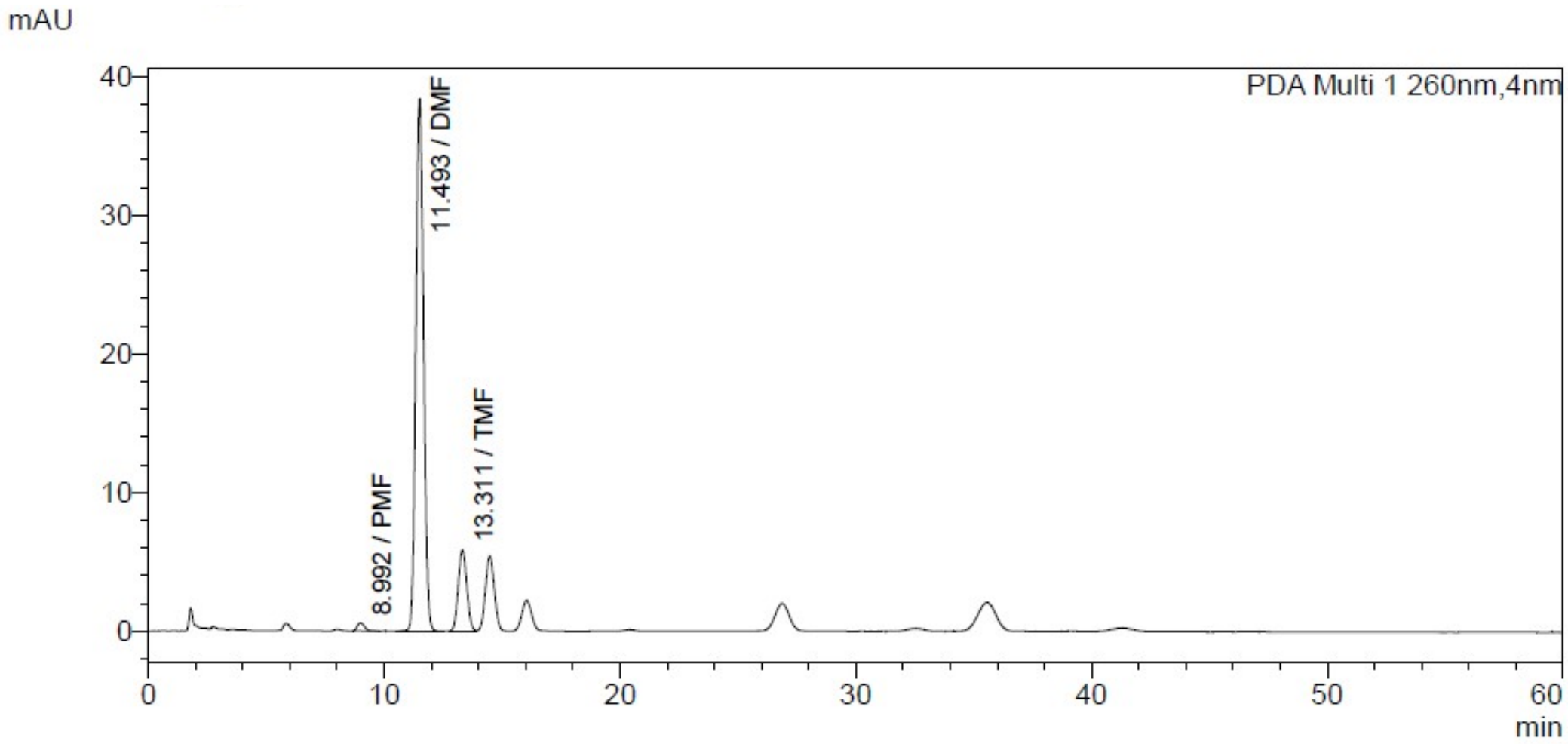

<Peak Table>

PDA Ch1 260nm

Peak#	Ret. Time	Area	Height	Conc.	Unit	Mark	Name
1	8.992	12962	595	0.000	mg/L	M	PMF
2	11.493	878585	38403	0.000	mg/L	M	DMF
3	13.311	147891	5844	0.000	mg/L		TMF
Total		1039438	44842				

Figure S3. UPLC of major polymethoxyflavones (PMFs) such as 5,7-dimethoxyflavone (DMF), 5,7,4'-trimethoxyflavone (TMF), and 3,5,7,3',4'-pentamethoxyflavone (PMF) at 20 mg/L

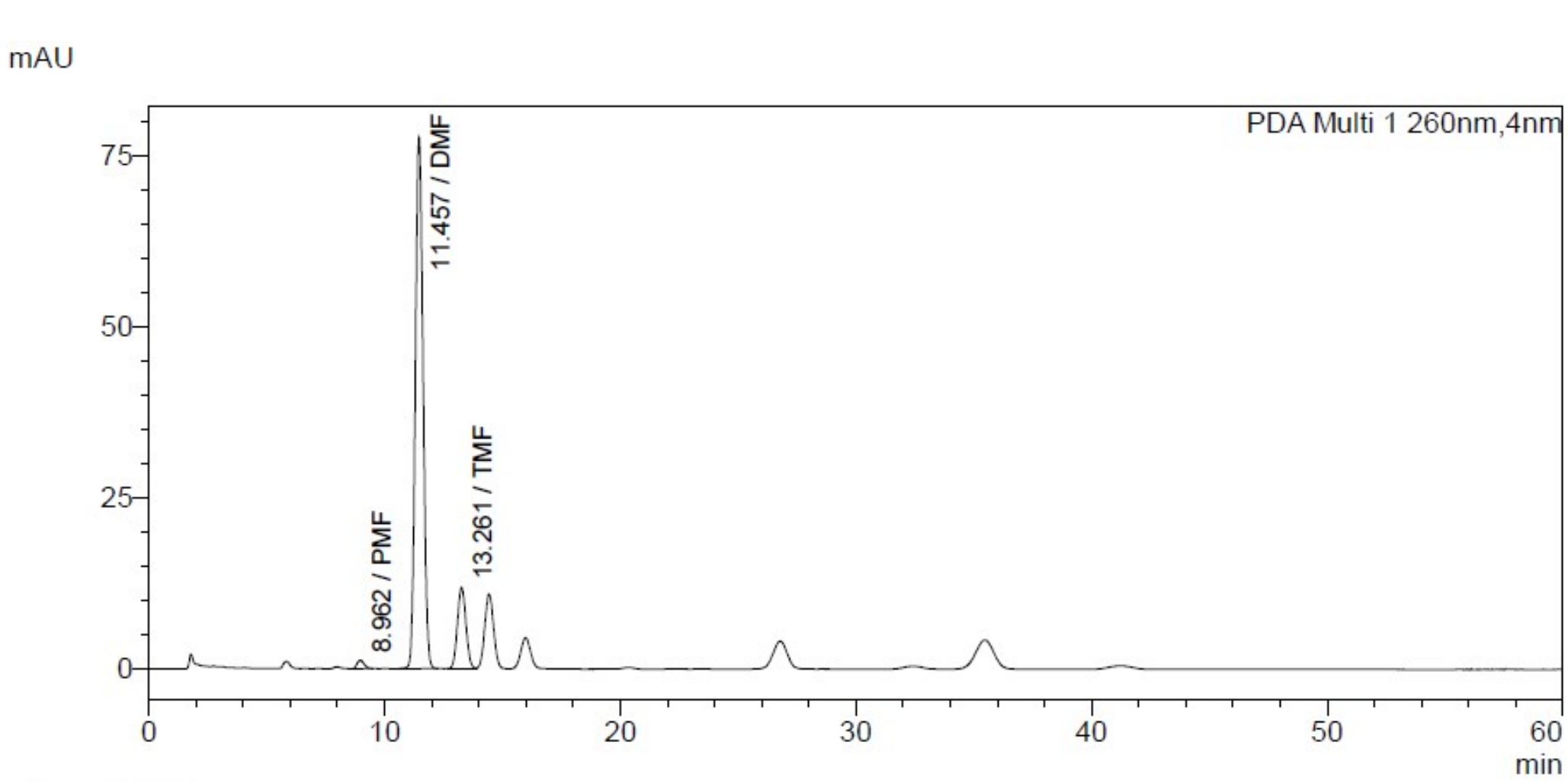

<Peak Table>

PDA Ch1 260nm

Peak#	Ret. Time	Area	Height	Conc.	Unit	Mark	Name
1	8.962	25329	1215	0.000	mg/L		PMF
2	11.457	1766166	77645	0.000	mg/L	M	DMF
3	13.261	298940	11862	0.000	mg/L		TMF
Total		2090435	90721				

Figure S4. UPLC of major polymethoxyflavones (PMFs) such as 5,7-dimethoxyflavone (DMF), 5,7,4'-trimethoxyflavone (TMF), and 3,5,7,3',4'-pentamethoxyflavone (PMF) at 40 mg/L

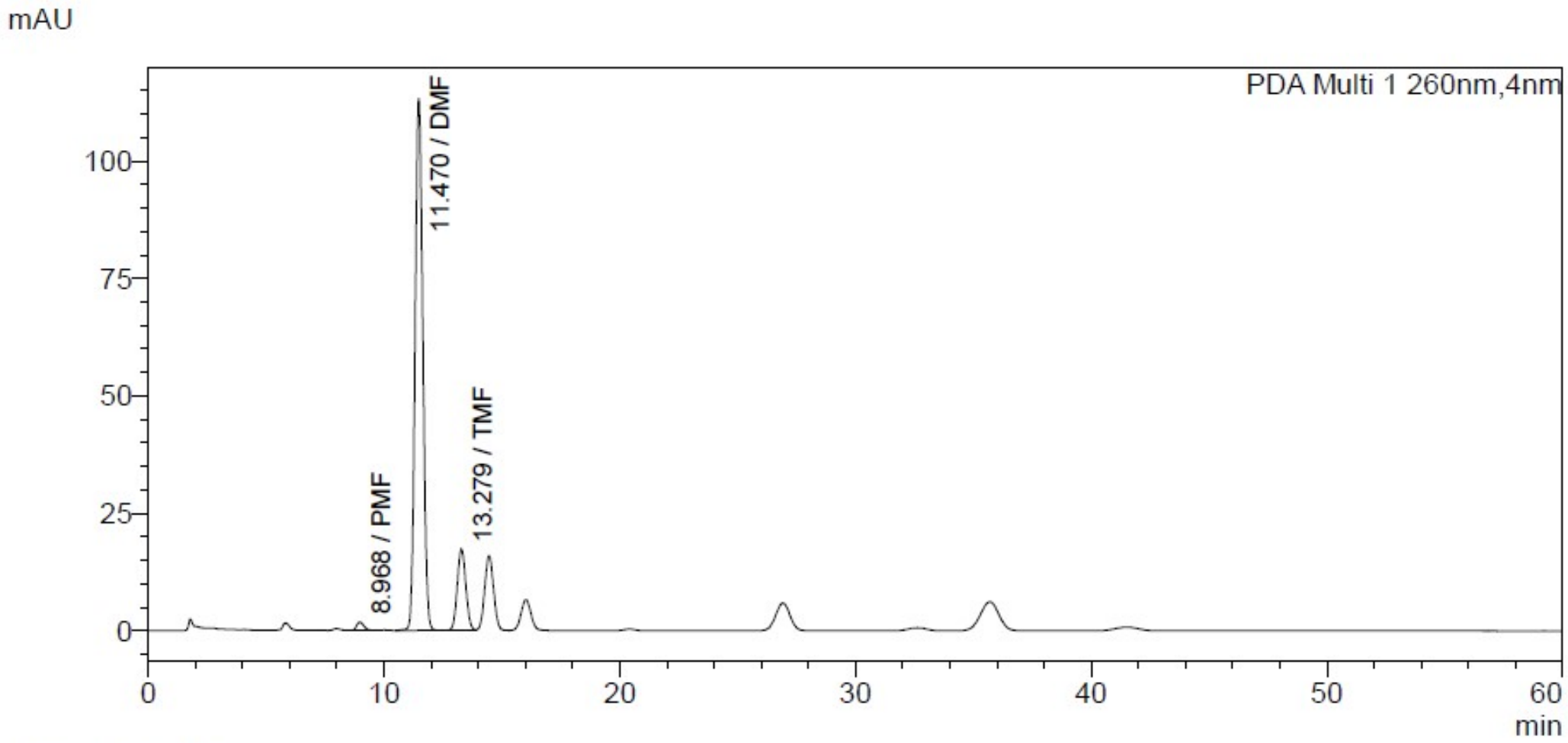

<Peak Table>

PDA Ch1 260nm

Peak#	Ret. Time	Area	Height	Conc.	Unit	Mark	Name
1	8.968	36345	1739	0.000	mg/L		PMF
2	11.470	2581592	113339	0.000	mg/L	M	DMF
3	13.279	437126	17299	0.000	mg/L		TMF
Total		3055062	132377				

Figure S5. UPLC of major polymethoxyflavones (PMFs) such as 5,7-dimethoxyflavone (DMF), 5,7,4'-trimethoxyflavone (TMF), and 3,5,7,3',4'-pentamethoxyflavone (PMF) at 60 mg/L

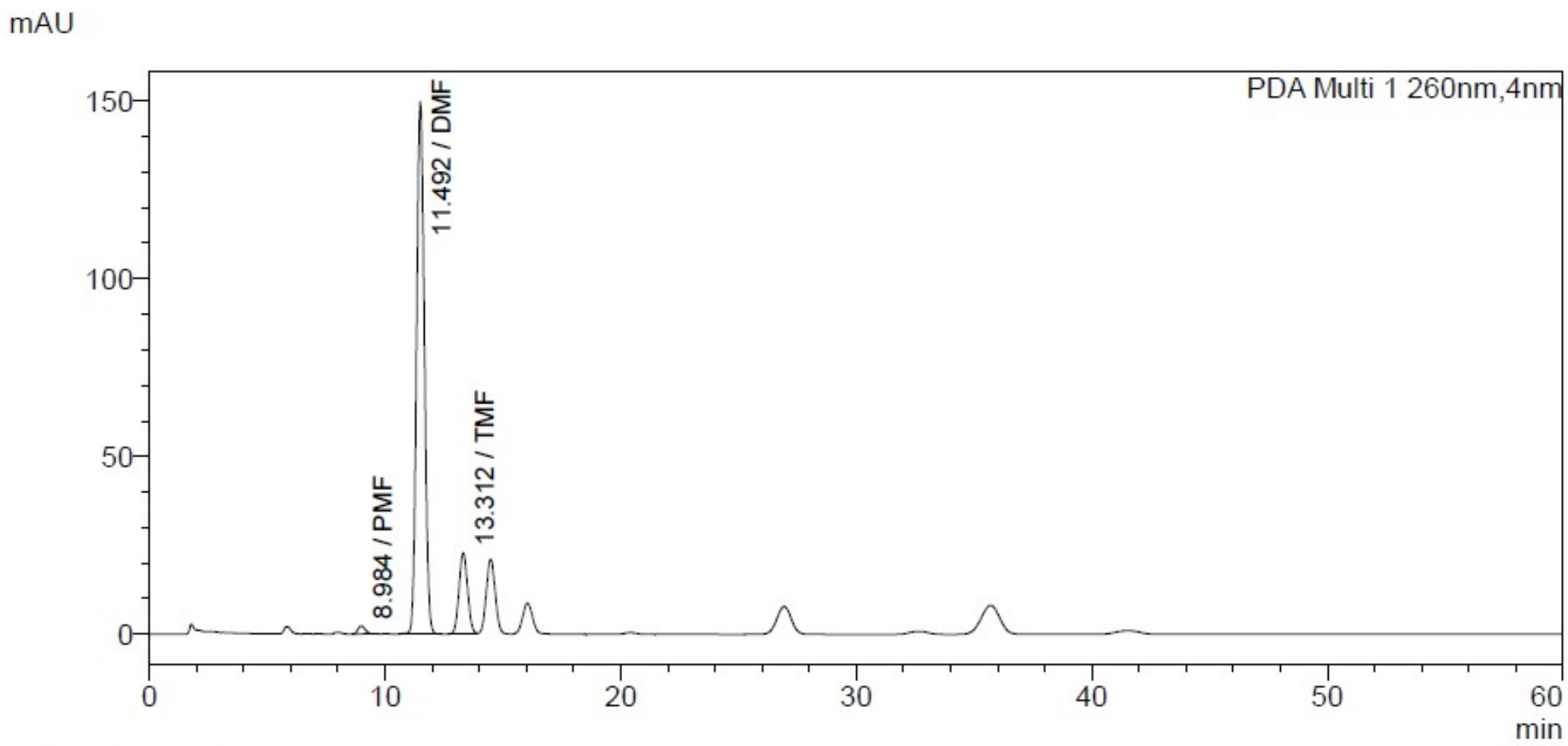

<Peak Table>

PDA Ch1 260nm

Peak#	Ret. Time	Area	Height	Conc.	Unit	Mark	Name
1	8.984	48212	2295	0.000	mg/L		PMF
2	11.492	3426346	149666	0.000	mg/L		DMF
3	13.312	580476	22811	0.000	mg/L		TMF
Total		4055034	174772				

Figure S6. UPLC of major polymethoxyflavones (PMFs) such as 5,7-dimethoxyflavone (DMF), 5,7,4'-trimethoxyflavone (TMF), and 3,5,7,3',4'-pentamethoxyflavone (PMF) at 80 mg/L

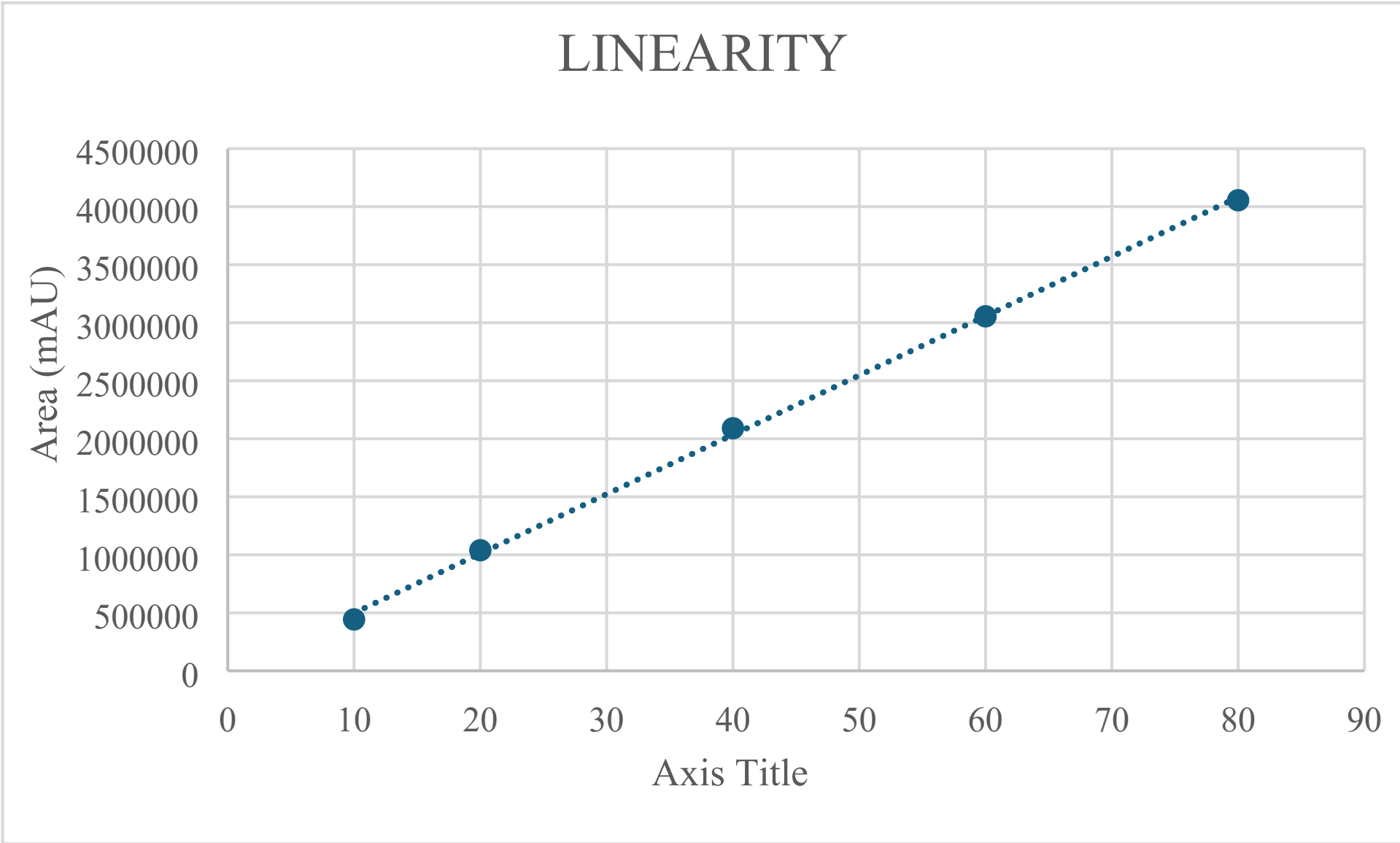

Concentration (mg/L)	Area (mAU)
10	443174
20	1039438
40	2090435
60	3055062
80	4055034

Figure S7. Calibration curve generated from mixed-standard injections containing 5,7-dimethoxyflavone (DMF), 5,7,4′-trimethoxyflavone (TMF), and 3,5,7,3′,4′-pentamethoxyflavone (PMF) at concentrations of 10–80 mg/L. For each concentration level, the summed peak area (DMF + TMF + PMF) was used to construct the total PMF calibration curve (Y=51174x - 12700; $R^2 = 0.9991$).

All validation, sample analysis, and stability study concentrations were within this validated calibration range, and no extrapolation beyond the calibration limits was applied.

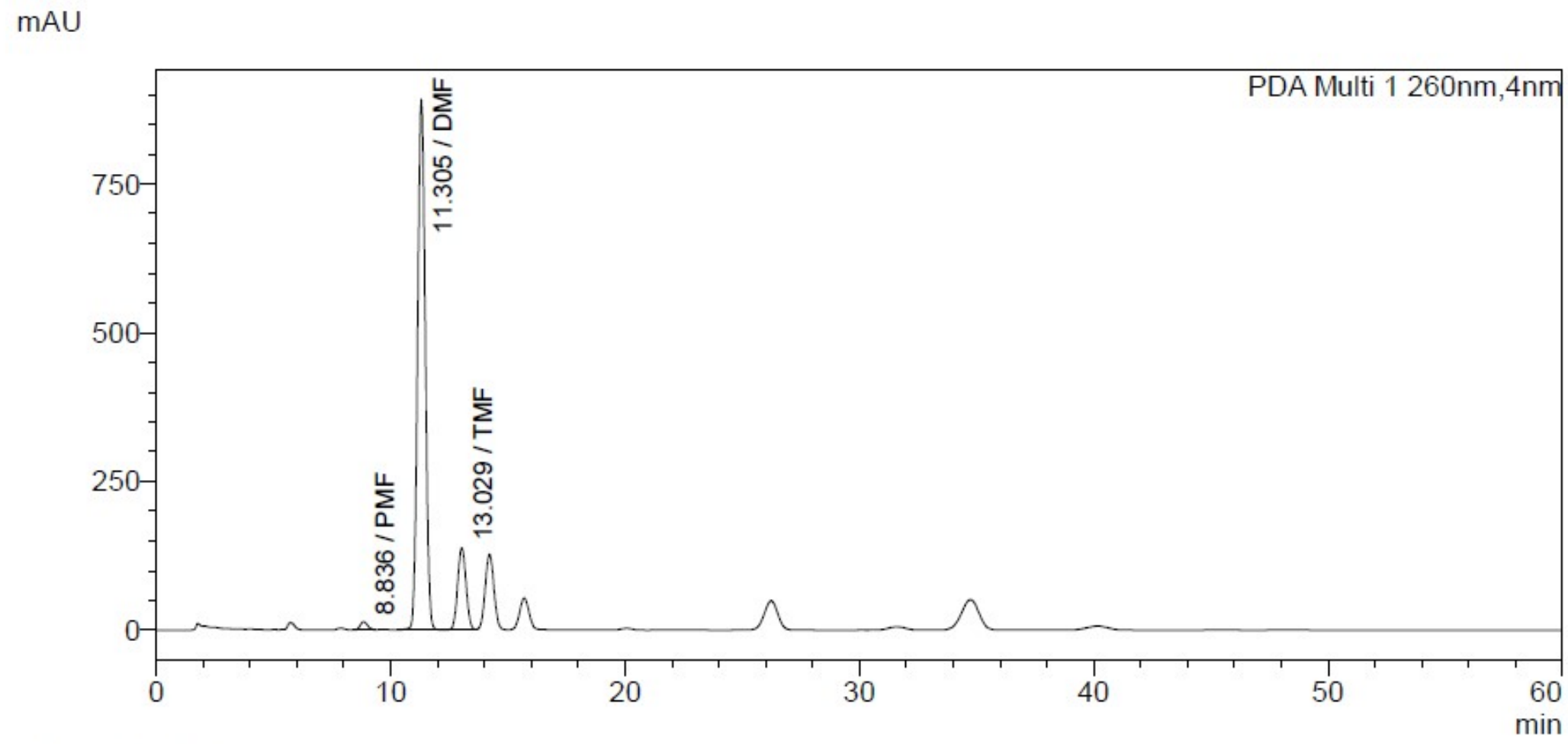

<Peak Table>

PDA Ch1 260nm

Peak#	Ret. Time	Area	Height	Conc.	Unit	Mark	Name
1	8.836	298879	13631	0.000	mg/L		PMF
2	11.305	20213820	890479	0.000	mg/L		DMF
3	13.029	3455877	138126	0.000	mg/L	V	TMF
Total		23968576	1042235				

Figure S8. UPLC chromatogram of *K. parviflora* extract

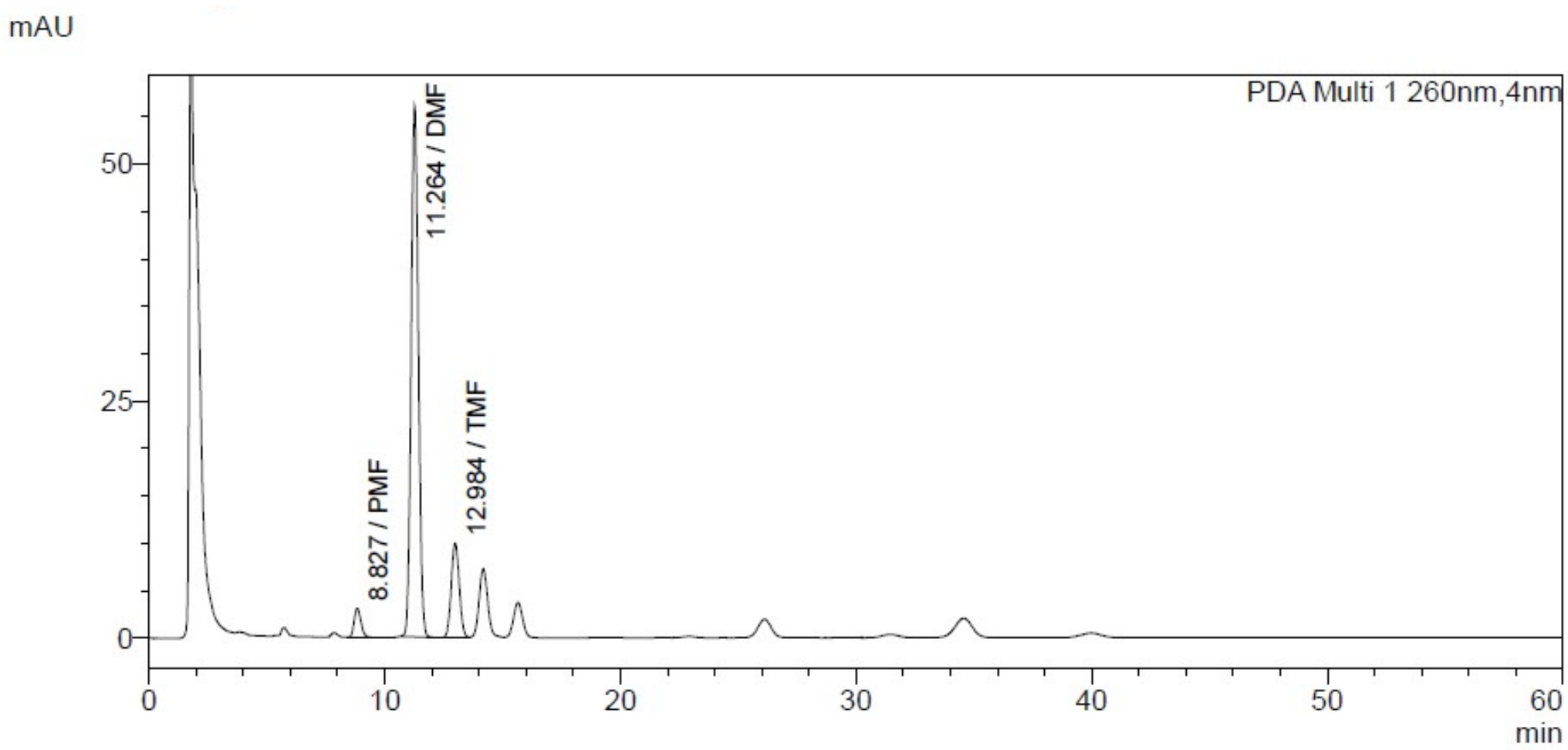

<Peak Table>

PDA Ch1 260nm

Peak#	Ret. Time	Area	Height	Conc.	Unit	Mark	Name
1	8.827	60872	3057	0.000	mg/L		PMF
2	11.264	1193562	56015	0.000	mg/L		DMF
3	12.984	235498	9959	0.000	mg/L		TMF
Total		1489932	69031				

Figure S9. UPLC chromatogram of Testolift

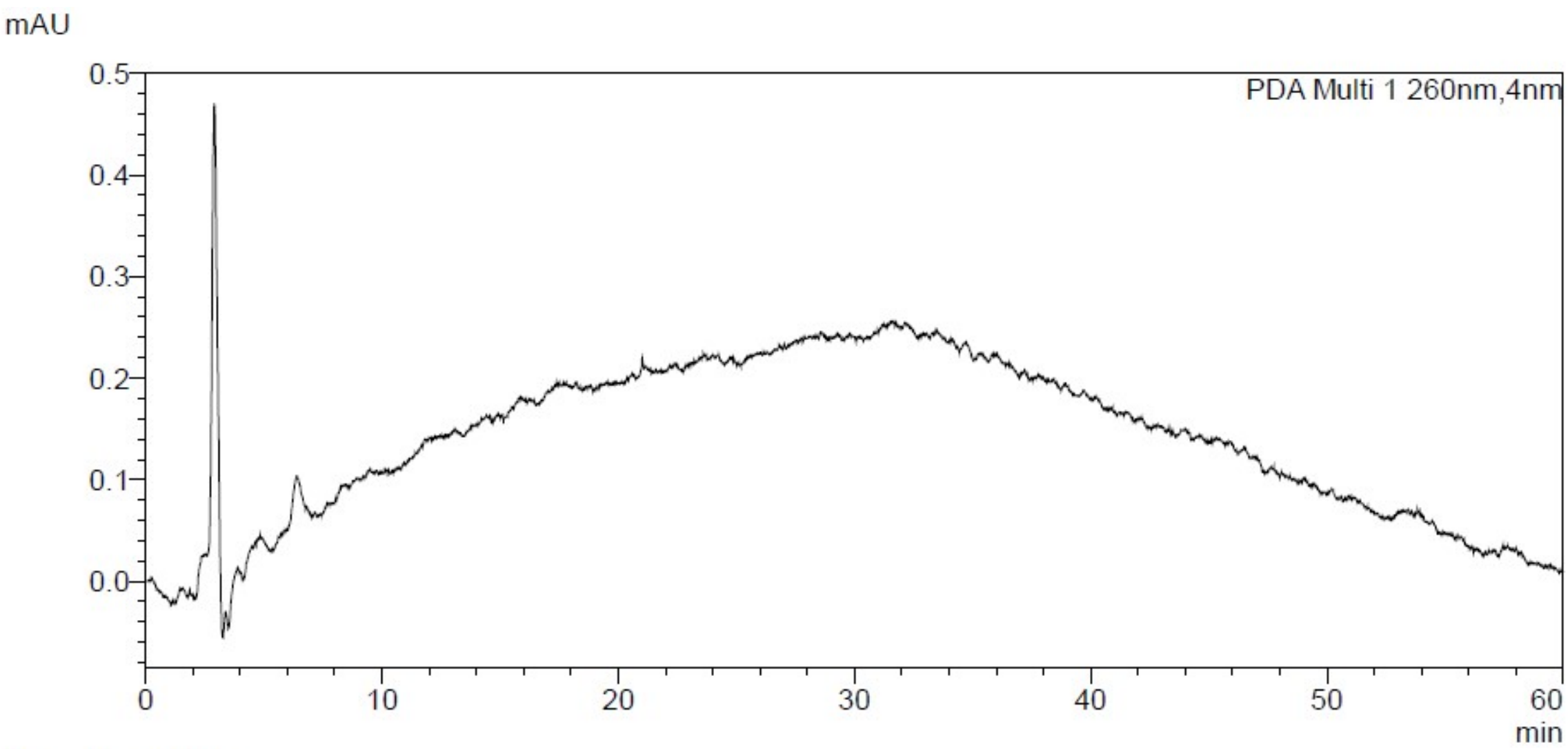

<Peak Table>

PDA Ch1 260nm

Peak#	Ret. Time	Area	Height	Conc.	Unit	Mark	Name
Total							

Figure S10. UPLC chromatogram of blank

Sample quantification of PMFs was performed using the finalized calibration curve (10–80 mg/L; slope = 51174; intercept = −12700; R^2 = 0.9991)

S3. Analytical Method Validation for Polymethoxyflavones (PMFs)

S3.1. Repeatability

Repeatability was determined by analyzing five replicate injections (n=5) of the standard solution at a concentration of 10 mg/L.

Table S2. Repeatability Data for Polymethoxyflavones (PMFs)

Injection No.	Compound Name	Spike Level (mg/L)	Peak Area (mAU)
1	PMFs	10	438924
2	PMFs	10	440087
3	PMFs	10	443174

4	PMFs	10	442644
5	PMFs	10	452136
Mean			443493
Standard Deviation (SD)			5193.9
% Relative Standard Deviation (% RSD)			1.171

Results: The mean peak area was 443,493 mAU, with a Standard Deviation (SD) of 5193.9 mAU. The calculated % RSD was 1.171%, which is below the acceptance limit of ≤ 2%, confirming the excellent precision of the method.

S3.2. Accuracy

Accuracy was determined by performing five replicate spike additions (n=5) at three different concentration levels: 10 mg/L, 20 mg/L, and 40 mg/L.

Table S3. Accuracy Determination for PMFs via Spike Recovery

Spike level (mg/L)	Replicate	Measure Concentration (mg/L)	% Recovery	Mean Recovery (%)
10	1	8.584784	85.85	86.74
	2	8.608001	86.08	
	3	8.669627	86.70	
	4	8.659047	86.59	

	5	8.848536	88.49	
20	1	19.49201	97.46	98.81
	2	19.63581	98.18	
	3	19.7586	98.79	
	4	19.97064	99.85	
	5	19.95768	99.79	
40	1	39.9772	99.94	100.05
	2	39.99955	100.00	
	3	40.03703	100.09	
	4	40.03293	100.08	
	5	40.05368	100.13	

Results: The mean recovery percentages were 86.74% (10 mg/L), 98.81% (20 mg/L), and 100.05% (40 mg/L). All mean recovery results fell within the acceptable range of 80.0% - 120.0%, demonstrating the accuracy of the method.

S3.3. Precision

The precision of the method was assessed by performing five replicate injections (n=5) of spiked samples at four different concentration levels (10, 20, 40, and 60 mg/L).

Table S4. Precision Study for PMFs over a Concentration Range

Spike Level (mg/L)	Replicate	Concentration Found (mg/L)	% RSD
10	1	8.584784	1.19%
	2	8.608001	
	3	8.669627	
	4	8.659047	

	5	8.848536	
20	1	19.49201	1.05%
	2	19.63581	
	3	19.7586	
	4	19.97064	
	5	19.95768	
40	1	39.9772	0.08%
	2	39.99955	
	3	40.03703	
	4	40.03293	
	5	40.05368	
60	1	59.46124732	0.95%
	2	58.91822957	
	3	59.8415566	
	4	60.26310176	
	5	60.25077816	

Results: The precision expressed as the percentage Relative Standard Deviation (% RSD) of the concentration found, ranged from 0.08% to 1.05%. All % RSD values were less than the acceptance criterion of ≤2%, confirming the satisfactory precision of the analytical method across the tested range.

S3.4. Limit of Detection (LOD) and Limit of Quantification (LOQ)

The LOD and LOQ were determined by the signal-to-noise (S/N) ratio method. Standard solutions of PMFs were prepared at decreasing concentrations and injected to determine the level that produced a peak response with an S/N ratio of approximately 3 for LOD and 10 for LOQ.

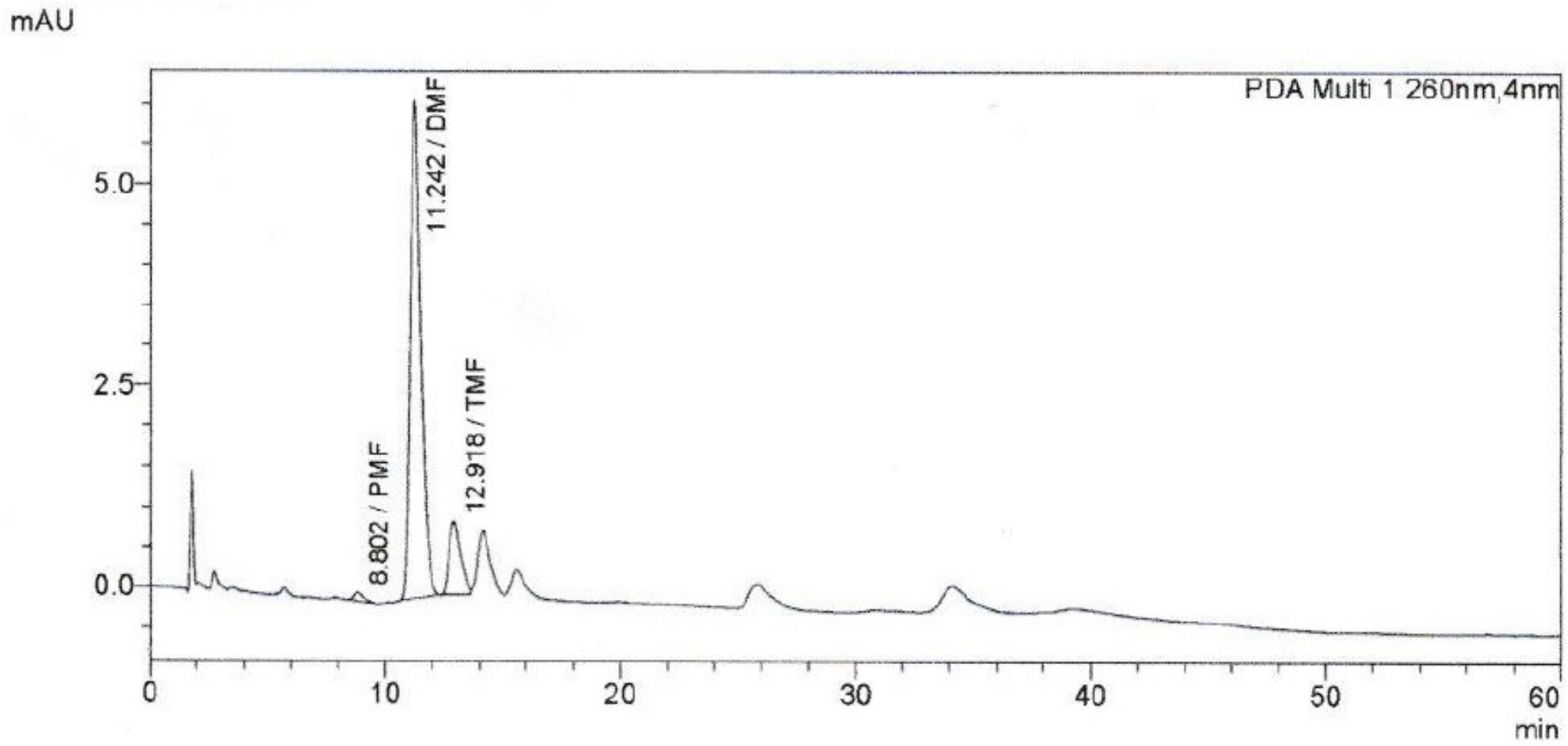

<Peak Table>

PDA Ch1 260nm

Peak#	Ret. Time	Area	Height	Conc.	Unit	Mark	Name
1	8.802	2938	102	0.000	mg/L	M	PMF
2	11.242	198445	6190	0.000	mg/L		DMF
3	12.918	31499	918	0.000	mg/L		TMF
Total		232883	7210				

Figure S11. Chromatogram for the determination of the Limit of Detection (LOD) of PMFs at 5 mg/L

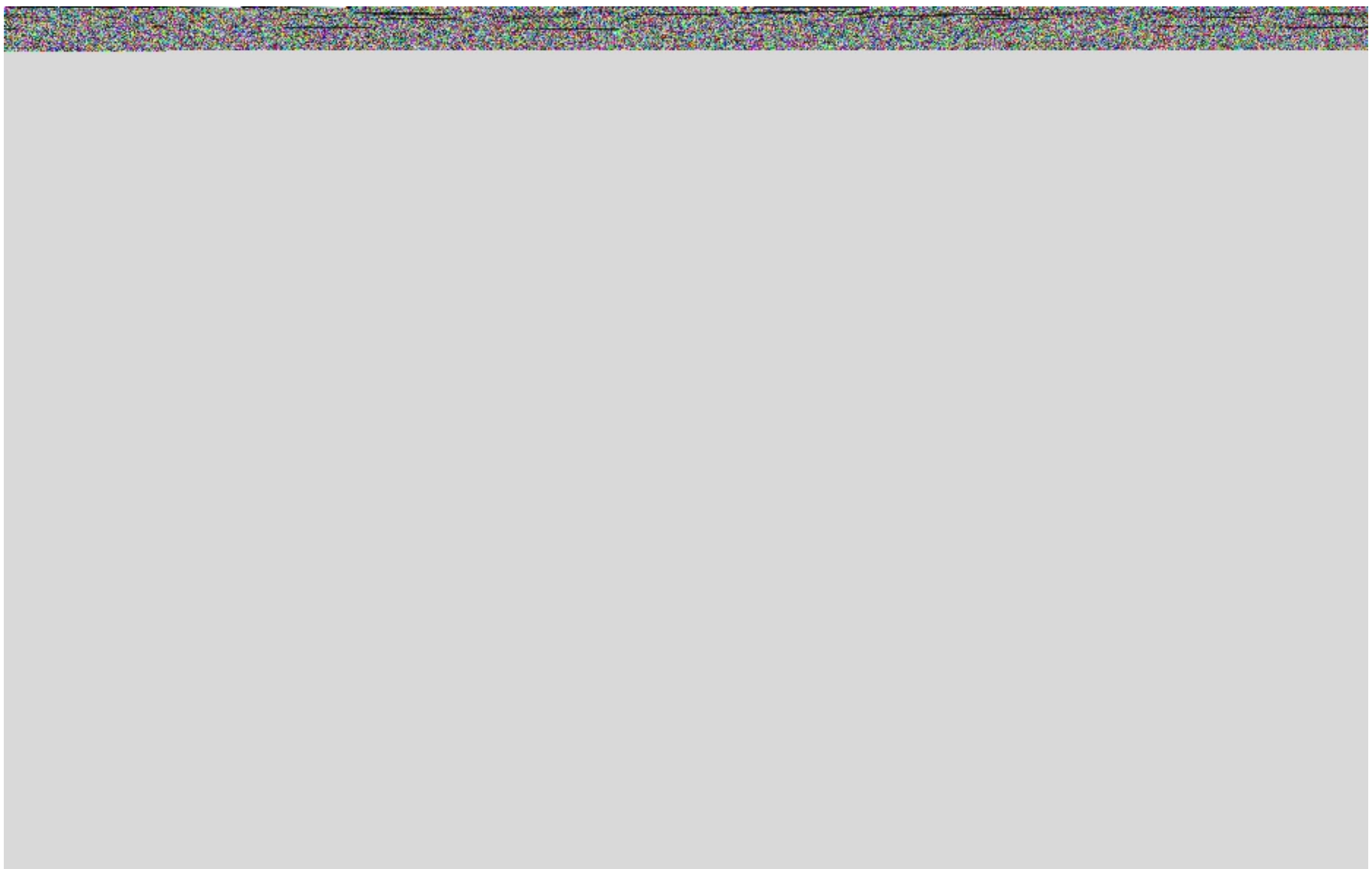

Figure S12. Chromatogram for the determination of the Limit of Quantification (LOQ) of PMFs at 10 mg/L

Results: The LOD was established at a concentration of 5 mg/L for all three PMFs, as shown in the chromatogram (Figure S11), corresponding to an S/N ≈3. The LOQ was established at a concentration of 10 mg/L (Figure S12), corresponding to an S/N≈10, confirming the sensitivity of the method.
